# Long-term patterns of abundance, residency and movements of bull sharks (*Carcharhinus leucas*) in Sydney Harbour, Australia

**DOI:** 10.1038/s41598-019-54365-x

**Published:** 2019-12-11

**Authors:** Amy F. Smoothey, Kate A. Lee, Victor M. Peddemors

**Affiliations:** 1 NSW Department of Primary Industries, Fisheries Research, Sydney Institute of Marine Science, Mosman, NSW 2088 Australia; 2 Sydney Institute of Marine Science, Mosman, NSW 2088 Australia

**Keywords:** Animal migration, Behavioural ecology, Conservation biology, Urban ecology

## Abstract

Bull sharks (Carcharhinus leucas) are known to frequent nearshore environments, particularly estuaries, resulting in interactions with humans. Knowledge of the behaviour of large individuals in temperate, estuarine environments is limited. This acoustic telemetry study reports on residency and movement patterns of 40 sub-adult and adult bull sharks in Sydney Harbour, a large temperate estuary, over seven years. Bull sharks exhibited clear seasonal patterns in their occurrence during the austral summer and autumn, with abundance peaking in January and February. This pattern was consistent between sexes and across all sizes. Bull sharks displayed weak diel differences in their spatial distribution, with individuals using areas further from the Harbour entrance more frequently during the day and at low tides. A diel pattern in depth use was apparent, with sharks utilising deeper water during daytime and moving shallower at night. Bull sharks had high individual inter-annual variability in their spatial distribution, however, when data were aggregated among all individuals and years, two locations of increased use were identified. Water temperature was the key predictor for seasonal movements and return behaviour to this estuary, suggesting that increasing water temperatures as a result of climate change may lead to higher shark abundance and possibly longer periods of residency in Sydney Harbour. Understanding the drivers for bull shark abundance and distribution will hopefully facilitate better education and shark smart behaviour by estuarine water-users, especially during summer and autumn months.

## Introduction

Many species of sharks are high-order predators and play important roles in structuring marine communities, either directly or indirectly^1–4^. With several populations declining throughout the world due to habitat-loss and alteration, coupled with the threats from over-fishing^5,6^, there is an urgent need to understand aspects of their biology, ecology and behaviour to enable effective management of populations and, increasingly, for reducing potentially hazardous shark-human interactions.

Despite the world-wide trend of unprovoked shark bites increasing in frequency^7^, these events are still relatively rare. Yet, when they occur they receive large amounts of media attention, concern from the public^8^, and significant political interest which has led to the implementation of shark bite mitigation strategies to increase public safety^9^. Historically, bather protection programs have focused on lethal mitigation measures^10^, however, attention has shifted recently towards non-lethal mitigation strategies such as, catch and relocate approaches^11^, aerial^12,13^ or land-based surveillance^14^ and increasing our understanding of the ecology of potentially dangerous sharks (e.g. white sharks, *Carcharodon carcharias*^15^). Knowledge of their behaviour will enhance the predictability of shark encounters and thereby potentially reduce the risk of shark bites either through establishing: (i) education programs to modify human behaviour, in areas and times of increased risk^16^ or, (ii) mitigation strategies that are more target-specific.

In south-east Australia, shark interactions occur in nearshore waters with a distinct seasonal peak between November and April^17^. The impact of this seasonal increase was highlighted during the summer and autumn of 2009, with 13 unprovoked interactions occurring in NSW, including a particularly severe bite by a bull shark (*Carcharhinus leucas*) on a diver in Sydney Harbour. Consequently, the NSW Government requested information on the spatial and temporal patterns of distribution and abundance of sharks in Sydney Harbour. It was subsequently shown that bull sharks were the only potentially dangerous species of shark caught consistently throughout Sydney Harbour in summer and autumn^18^, but the processes governing these patterns required further investigation. Furthermore, water-users are often warned to avoid the water during crepuscular and nocturnal periods to reduce risks of encountering sharks, as these are the periods historically assumed to be times of increased activity and feeding. Here, we evaluate these assumptions by investigating diel differences in behaviour and use of habitat.

Bull sharks are large up to 4 m total length^19^ apex predators, globally distributed throughout rivers, estuaries, nearshore areas and continental shelf waters of tropical and sub-tropical regions. They are one of the few species of shark physiologically capable of tolerating freshwater^20^. Consequently, such tolerance enables them to frequent nearshore areas where they may interact more with water-users than other shark species. Studies done on the presence and movement of bull sharks have shown that their behaviours are complex and the propensity of individuals to move or stay, varies across different spatial and temporal scales and changes according to size and sex of the individual^21,22^. It is well documented that bull sharks, use estuarine and riverine environments, however, most of that work has been done on juveniles in tropical regions^23–27^. Their movements within those systems has been shown to be influenced by various environmental conditions such as, temperature^28^, salinity^23,29^, dissolved oxygen^30^ and rainfall^31^. Our understanding of the spatial ecology and behavioural patterns of adult bull sharks is still relatively limited, particularly in temperate waters. Recent work reported that adults migrate large distances, yet exhibit strong site fidelity on a seasonal or annual basis^21,32–35^. However, much remains unclear about the long-term space use, seasonal trends in movements and environmental drivers for habitat-use of adult bull sharks in temperate estuaries. Here, we report on the use of acoustic telemetry to investigate the spatial ecology of bull sharks in Sydney Harbour, specifically to examine: (i) whether there are diel and/or seasonal patterns, (ii) how long bull sharks spend in Sydney Harbour, (iii) if there are any areas of increased use, (iv) water depths used, and (v) whether these patterns of occurrence are influenced by environmental and biological drivers. This study provides essential knowledge of bull shark behaviour that will, ultimately enhance the possibility to more accurately predict the likelihood of bull sharks occuring in temperate waterways and thus contribute to an objective assessment of the likelihood of bull sharks interacting with humans in these locales.

## Results

**Figure 1:**
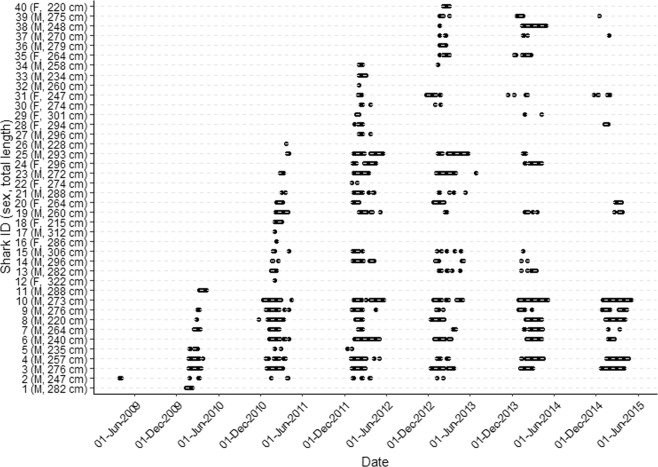
Presence-absence plot of bull sharks acoustically detected in Sydney Harbour from 2009 to 2015.

Forty bull sharks (12 females and 28 males) were tagged in Sydney Harbour ranging in size from 220 to 322 cm TL (Table 1). All sharks were classified as adults or sub-adults based on their size and/or the calcification of male claspers. Four sharks were only ever detected in the array on a single day post release (#12, 16, 26 and 32; male = 2, female = 2, Fig. 1) with the remaining sharks detected for varying lengths of time.

**Table 1:** Details of the 40 bull sharks tagged and detected in Sydney Harbour, New South Wales. Total length is the length at tagging.

Shark ID	Date tagged	Sex	TL (cm)	No. of days detected	No. of days monitored	Residency index	No. of years subsequently returned
1	4/03/09	Male	282	28	2277	0.01	0 (6)
2	24/03/09	Male	247	32	2257	0.01	4 (6)
3	21/01/10	Male	276	380	1954	0.19	4 (5)
4	21/01/10	Male	257	256	1954	0.13	5(5)
5	25/01/10	Male	235	13	1950	0.01	5(5)
6	8/02/10	Male	240	137	1936	0.07	5(5)
7	11/02/10	Male	264	144	1933	0.07	5(5)
8	17/02/10	Male	220	300	1927	0.16	5 (5)
9	24/02/10	Male	276	181	1920	0.09	5 (5)
10	2/03/10	Male	273	368	1914	0.19	5 (5)
11	3/03/10	Male	288	9	1913	0.00	0 (5)
12	11/01/11	Female	322	1	1599	0.00	0 (4)
13	11/01/11	Male	282	34	1599	0.02	2 (4)
14	11/01/11	Male	296	49	1599	0.03	3 (4)
15	19/01/11	Male	306	23	1591	0.01	3 (4)
16	19/01/11	Female	286	1	1591	0.00	0 (4)
17	25/01/11	Male	312	3	1585	0.00	0 (4)
18	27/01/11	Female	215	29	1583	0.02	0 (4)
19	1/02/11	Male	260	60	1578	0.04	4 (4)
20	4/02/11	Female	264	121	1575	0.08	3 (4)
21	12/02/11	Male	288	38	1567	0.02	2 (4)
22	12/02/11	Female	274	4	1567	0.00	1 (4)
23	21/02/11	Male	272	121	1558	0.08	2 (4)
24	3/03/11	Female	296	68	1548	0.04	2 (4)
25	8/03/11	Male	293	160	1543	0.10	3 (4)
26	11/03/11	Male	228	1	1540	0.00	0 (4)
27	6/04/11	Male	296	4	1514	0.00	0 (3)
28	9/01/12	Female	294	24	1236	0.02	1 (3)
29	18/01/12	Female	301	17	1227	0.01	1 (3)
30	24/01/12	Female	274	13	1221	0.01	1 (3)
31	25/01/12	Female	247	70	1220	0.06	3 (3)
32	30/01/12	Male	260	1	1215	0.00	0 (3)
33	31/01/12	Male	234	26	1214	0.02	0 (3)
34	31/01/12	Male	258	18	1214	0.01	1 (3)
35	16/01/13	Female	264	61	863	0.07	1 (2)
36	16/01/13	Male	279	27	863	0.03	0 (2)
37	17/01/13	Male	270	9	862	0.01	2 (2)
38	17/01/13	Male	248	92	862	0.11	1 (2)
39	17/01/13	Male	275	41	862	0.05	2 (2)
40	30/01/13	Female	220	21	849	0.02	0 (2)

Number inside brackets represents the maximum number of years monitored in this study.

### Abundance and residency

**Figure 2:**
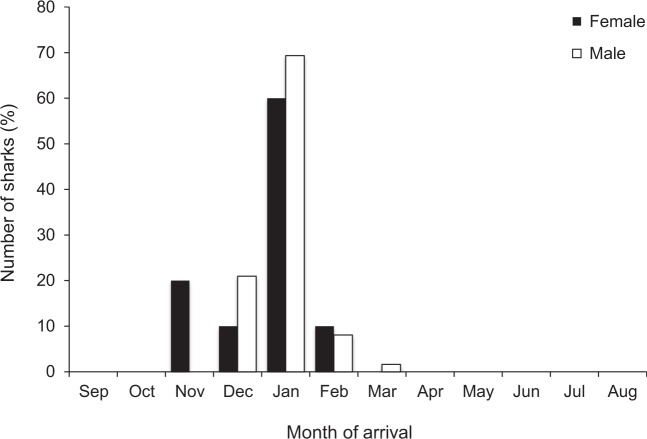
Month that sharks arrived in Sydney Harbour (across all years) by sex.

**Figure 3:**
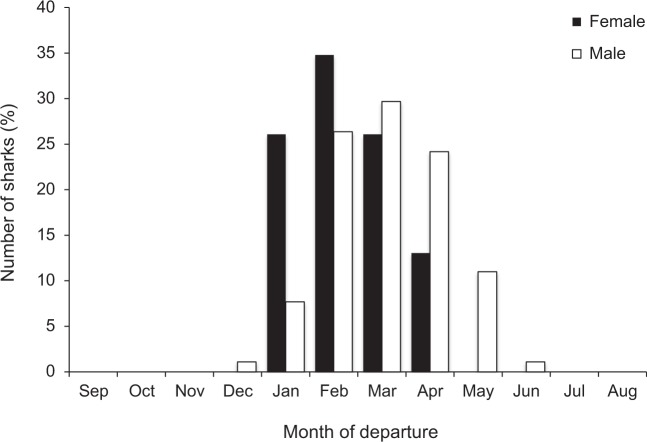
Month that sharks departed from Sydney Harbour (across all years) by sex.

The number of days per year that sharks were detected in the Harbour over the entire study ranged from 1 to 105 days (26.7 ± 1.8; mean ± SE) and was higher for males (30.7 ± 2.2) than females (18.6 ± 2.2). Residency of bull sharks in Sydney Harbour ranged from 0 to 0.19 (Table 1) and did not differ between males (0.05 ± 0.01) and females (0.02 ± 0.01; T-test, t = 1.38, df = 38, p > 0.05). Throughout the study, 70% of tagged sharks subsequently returned to the Harbour on one or more years post tagging and 28% of individuals returned on all possible years. Despite there being no significant differences in the time of arrival or departure between sexes, among sharks of different sizes, among years or for the sex-size interaction (MCMCglmm, all p-values > 0.05), some females arrived in Sydney Harbour earlier than males (Fig. 2) and some males departed earlier than females (Fig. 3). The highest proportion of females and males arrived in January (Fig. 2) and a high percentage of both sexes departed in February and March (Fig. 3), yet male bull sharks were present for one month longer than females.

**Figure 4:**
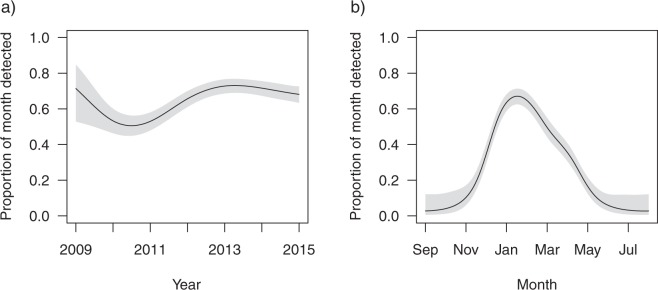
Response curves of the proportion of month that sharks were present in Sydney Harbour GAMM, showing the influence of (**a**) year and **(b**) month on the number of days individual sharks were detected per month. Shaded areas indicate the 95% confidence intervals.

The proportion of a month that sharks were detected showed significant inter-annual (Fig. 4a) and seasonal (Fig. 4b) variation but did not vary with size, sex or size according to sex (none of the latter variables were included in final model). Sharks spent less time in Sydney Harbour in 2010 and 2011 than the other years of the study (Fig. 4a). The model accounted for the varying number of receivers deployed in Sydney Harbour, with the largest array being deployed in 2011. The variation in detections between years was therefore not due to disparity in the number of receivers deployed. January and February represented the period when bull sharks spent the highest proportion of the month in Sydney Harbour, whilst they were completely absent between July and October (Fig. 4b). However, the final model only accounted for 42.7% of variation observed in the data suggesting other factors not considered in our models also influence how many days per month bull sharks were detected.

### Diel, tidal and depth

**Figure 5:**
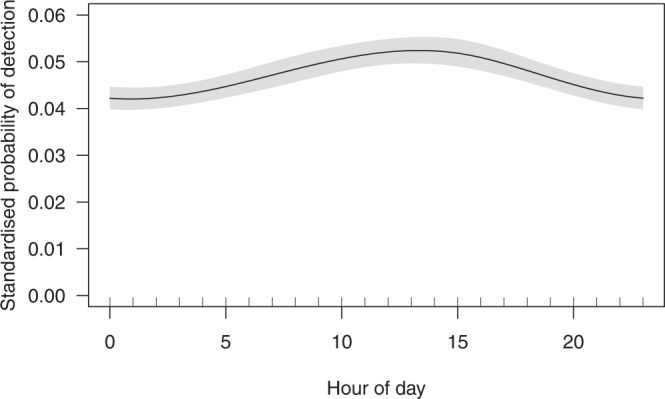
Response curve of standardised proportion of detections per hour GAMM, showing the influence of the hour of the day on the probability of detection. The proportion of detections per hour were standardised by the standardised detection frequencies (SDFs) calculated from sentinel tags.

**Figure 6:**
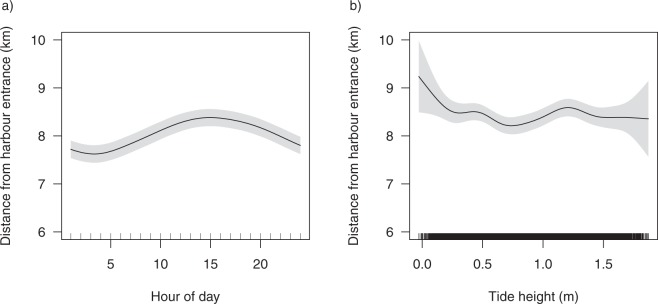
Generalised additive mixed model response curves showing the influence of (**a**) hour of the day and (**b**) the tidal height (m) on the distance of the centre of activity to the entrance of Sydney Harbour per hour.

Standardised probability of detection showed that sharks were detected more during daylight hours than at night (Fig. 5). However, there was only a 10% difference in the probability of detection between the peak (between 13:00 to 14:00; ~53% probability of being detected) and trough (midnight; ~43% probability of detection) and the model only accounted for 4.01% of the variability observed in the data. Sharks utilised habitats further from the Harbour entrance during the day than at night (Fig. 6a) and at low tide (Fig. 6b). However, the model indicated that sharks utilised distances of greater than 6 km from the Harbour entrance and only accounted for 2.68% of the variability in the data.

**Figure 7:**
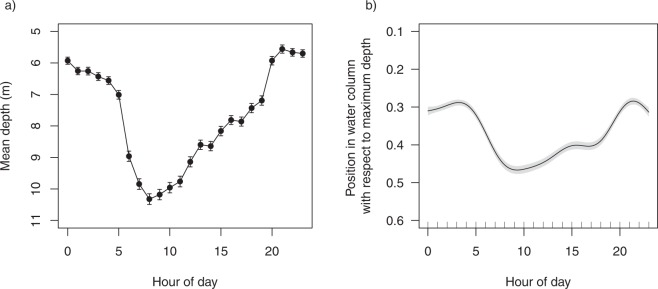
(**a**) Mean depth used by sharks for each hour of the day and (**b**) Generalised additive mixed model response curve showing the influence of hour of the day on the position in the water column that sharks were detected.

Depths used by sharks (n = 23) ranged from 0 to 40.02 m; with a mean of 7.59 ± 0.03 m. Sharks exhibited a diel pattern in their depth use, with individuals utilising slightly deeper positions during the day than at night (Fig. 7a,b). This model accounted for a higher amount of variability in the data than the previous two models (20.7%). The descent of tagged bull sharks into deeper water at daybreak, occupation of mean water depths greater than 7 m during daytime and moving shallower during twilight hours suggests strong diel patterns in habitat use (Fig. 7a).

### Area use of Sydney Harbour

**Figure 8:**
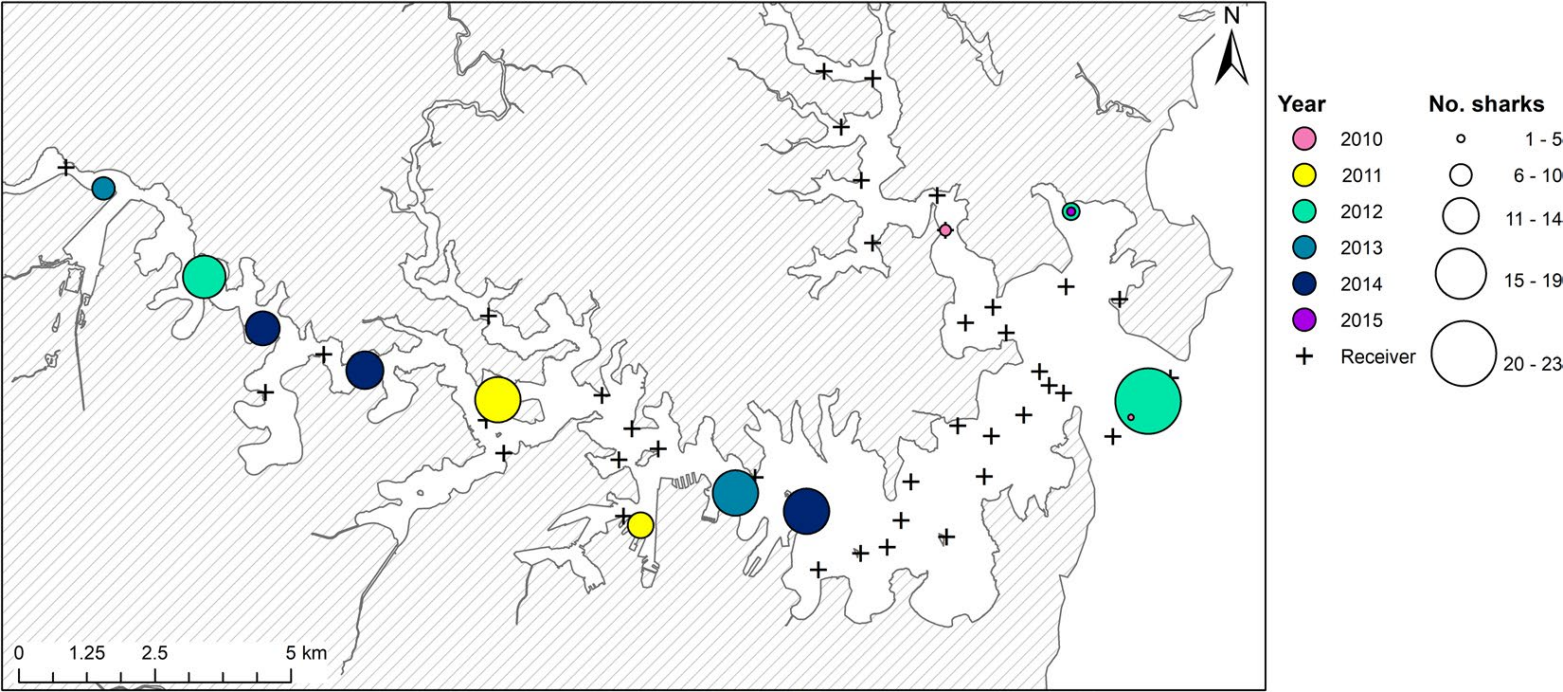
Annual variation in the distribution of ‘hotspots’ for shark detections in Sydney Harbour and number of sharks detected at each of these ‘hotspots’ as identified through spatial hotspot analysis.

**Figure 9:**
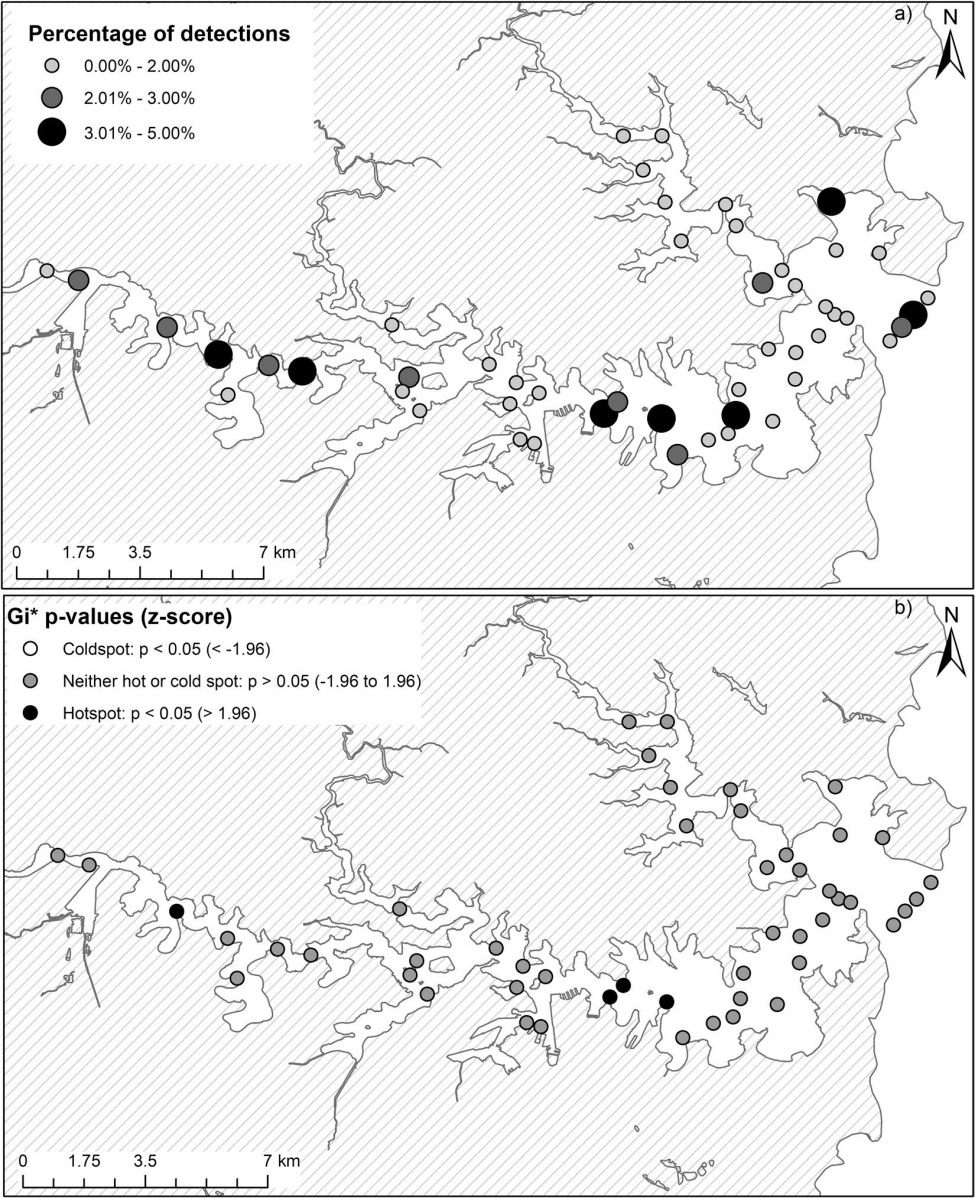
(**a**) The overall proportion of bull shark detections per receiver in Sydney Harbour and (**b**) the aggregate distribution of ‘hotspot’ versus ‘coldspot’ areas within these waters for all sharks throughout the study.

**Figure 10:**
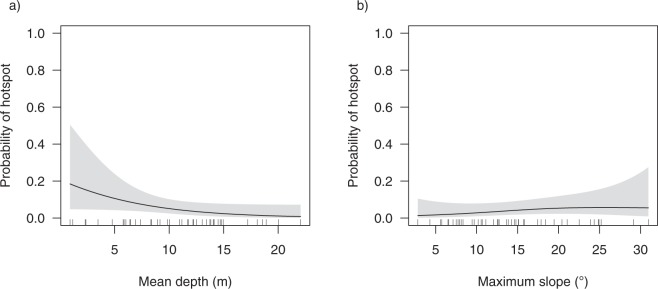
Influence of (**a**) mean depth and (**b**) maximum slope on the probability of a receiver being deployed within a shark ‘hotspot’.

Sharks were detected on all the receivers deployed in Sydney Harbour, however, there was high individual variability in the spatial distribution of bull sharks across the monitored area (Fig. 8). This led to inter-annual variation in the location of ‘hotspots’ (defined as areas of above the mean number of detections per day deployed), and in the total number of sharks detected at each of the receivers within these ‘hotspots’ (Fig. 8). When data were aggregated among all individuals and years (Fig. 9), no ‘coldspot’ (defined as areas below the mean number of detections per day deployed) areas were found within the array (Fig. 9b). Although, seven receivers had a higher percentage of detections (Fig. 9a), only two ‘hotspot’ locations were identified (Fig. 9b). The two areas of increased occurrence were: (i) the modified urban region immediately east of the Sydney Harbour Bridge (Garden Island, Opera House & Kirribilli) and (ii) Mortlake Point, at the entrance to the mangrove-lined Yaralla Bay in the Parramatta River. The probability that a location was a ‘hotspot’ was higher in mean water depths less than 5 m (Fig. 10a) and areas with steeper drop-offs (i.e. greater maximum slope; Fig. 10b). However, there was no influence by year, as this variable was not included in the final model structure.

### Environmental correlates to shark presence and abundance

**Figure 11:**
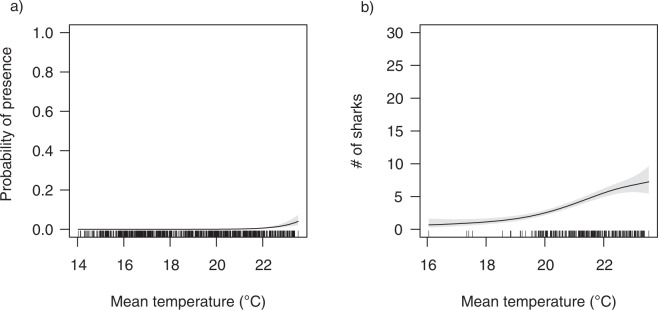
Generalised additive mixed model response curves showing the influence of (**a**) water temperature on daily shark presence-absence and (**b**) water temperature on shark abundance in Sydney Harbour.

Shark presence-absence and abundance were both only influenced by water temperature and not total rainfall, total rainfall the previous day or moon illumination. Probability of sharks being present increased when the water temperature was ~22°C (Fig. 11a) and this model accounted for 52.5% of the variability observed in the data. Similarly, the number of sharks detected increased in water temperatures from ~19 to 23°C (Fig. 11b) and this model accounted for 22.6% of the variability observed in the data.

## Discussion

Studies investigating the movements of sharks have increased in recent years, yet until now there was limited knowledge about the long-term patterns of abundance, residency and movements of large potentially dangerous sharks in heavily urbanised, temperate estuaries. Here, we demonstrated that Sydney Harbour, the most urbanised and iconic waterway in Australia^36^ is an important habitat for adult and sub-adult bull sharks. Acoustic telemetry data from seven years revealed that bull sharks exhibited distinct patterns of presence, abundance and residency through time. Bull sharks demonstrated clear seasonal patterns in their occurrence during the austral summer and autumn with abundance peaking in January and February across all years supporting earlier findings of increased catches during these months^18^.

The clear seasonal patterns in abundance and residency found in this study are consistent with previous studies done in sub-tropical and tropical environments. Daly *et al*.^34^ reported that bull sharks tagged on the east coast of southern Africa migrated south into more temperate latitudes during summer before migrating north to warmer latitudes during the austral winter and spring. These patterns were reflected in an increase in catch of bull sharks in bather-protection programs in higher latitudes off the east coast of South Africa during December, with a decline during austral winter and spring^37^. Conversely, researchers working in tropical latitudes report greater abundance in cooler months, with some sex-based difference in abundance and distribution^21,38^. In our study, we tagged twice as many males as females, yet while there were no sex-based differences in the time of arrival or departure and residency times, the number of days detected in the Harbour was greater for male bull sharks. In addition, the lack of heavily pregnant females and mating bites on sexually mature females caught during our study, suggests that this southern distribution of large bull sharks is unlikely related to reproductive activities but may be driven by foraging opportunities.

Residency indices for bull sharks reported here were sustainably lower than those values published previously^21,33,34^. This is likely due to the fact that Sydney Harbour is towards the southern extent of the distribution of bull sharks^39^ and whilst bull sharks are abundant in the Harbour throughout the austral summer and autumn, they were largely absent between June and November, which is correlated to their movement to lower latitudes^35^. Espinoza *et al*.^21^ reported that not all bull sharks from those lower latitudes participate in these large-scale movements, with a large proportion of the tagged sharks, predominately males, remaining in the study site year-round. Lee *et al*.^22^ highlighted the importance of estuaries as a key predictor for sub-adult and adult bull shark abundance along the east coast of Australia, yet interestingly found that bull sharks occurred more at mid-shelf habitats (20 to 60 m) compared to nearshore areas (<20 m water depth). Here, detections of tagged bull sharks decreased over the monitoring period, with the greatest decline occurring between 2010 and 2011. This decline could be explained by: (i) dispersal to areas without acoustic coverage, (ii) mortality associated with commercial and recreational fishing^40^, bather protection programs^41–44^, or natural mortality, and (iii) tag failure.

The distribution and habitat-use of sharks in nearshore environments have been shown to differ over various spatial and temporal scales as a result of variability in environmental conditions and/or biological drivers^18,29,30,45–48^. Throughout the austral summer and autumn when sharks were present in the Harbour, we found high individual variability in the spatial and temporal distribution of bull sharks, such that sharks were not homogeneously distributed throughout the Harbour over time. Although tagged sharks were detected on all receivers from the entrance to the upper river reaches, there were differences in: (i) the number of receivers visited by each individual shark, (ii) the number of sharks detected at each of the receivers and (iii) inter-annual variation in where ‘hotspots’ occurred. Interestingly, we found that when data were aggregated among all individuals and years, seven receivers interspersed throughout the Harbour had a higher percentage of detections through time, but only two locations were consistently identified as a ‘hotspot’. One area of increased occurrence was in the vicinity of Garden Island, the Opera House and Kirribilli. This area is a highly modified, urban region immediately east of the Sydney Harbour Bridge and was the location of an unprovoked bull shark bite early one morning in February 2009. The other area of increased bull shark utilisation was located at the entrance to the mangrove-lined Yaralla Bay in the Parramatta River. Whilst this area is still urbanised, it is less modified and the surrounding shores are fringed with mangroves^49^. Analysis of potential drivers for using these areas indicated that the best predictors of ‘hotspot’ locations were mean water depths being less than 5 m and areas with steeper drop-offs (i.e. greater maximum slope). It is unknown whether these habitat variables influence the behaviour of bull sharks directly or, indirectly by potentially influencing the availability of prey. In a recent teleost telemetry study in Sydney Harbour, Taylor *et al*.^50^ reported on the patterns of residency of several potential prey species. Although fine-scale temporal variation was not presented in their study, it was suggested that mulloway (*Argyrosomus japonicus*), silver trevally (*Pseudocaranx georgianus*) and yellow tail kingfish (*Seriola lalandi*) were resident throughout the year overlapping with the areas of increased occurrence of bull sharks through time. Furthermore, bottom topography in the form of deep holes or basins were identified as key habitats for mulloway residing in a temperate estuary south of Sydney Harbour^51^. It is therefore highly probable that bull sharks within the Harbour are more prevalent in areas of drop-offs and deep holes to optimise success of foraging. Further telemetry research of bull sharks and their prey in Sydney Harbour would potentially unravel this predator-prey relationship and assist in determining the drivers for habitat use in Sydney Harbour by bull sharks.

Many species alter their movement and use of habitat over smaller temporal scales to optimise opportunities for foraging or take advantage of prey availability^52^. In this study, we found weak diel differences in the patterns of movement of bull sharks, such that sharks were detected slightly more during the day than during periods of darkness and exhibited minor differences in their spatial distribution. Bull sharks tended to occupy areas further from the Harbour entrance during the day. Previous studies investigating diel patterns of activity in bull sharks have shown increased site fidelity during daylight hours followed by an absence or boarder use of habitat at night^53^. Similarly, Daly *et al*.^34^ reported that the majority of tagged adult bull sharks at the Pinnacle Reef in Mozambique exhibited different patterns in habitat-use over a 24-hour period and attributed these differences to reflect foraging behaviours. These diel differences in movements were not exhibited by bull sharks within Sydney Harbour waters, however, they did display diel differences in their use of the water column.

In this study, bull sharks in Sydney Harbour used slightly deeper water during the day and shallower water during night, a pattern also reported for adult bull sharks elsewhere^33,34^. In contrast, Carlson *et al*.^32^ found that sub-adult bull sharks tagged in coastal areas of the Gulf of Mexico and off the south-east coast of USA, did not exhibit differences in their depth usage between daytime and period of darkness, however, bull sharks in those areas exhibited a similar propensity for shallower waters as sharks in Sydney Harbour.

Studies examining the influence of lunar phase on abundance and movements of sharks have shown either direct or indirect effects of moon phase^54,55^. These patterns have often been attributed to moon-phase influencing the behaviour and abundance of predators as a result of illumination and/or tide, or indirectly, as a result of effects on the behaviour and distribution of prey. Moon illumination appears not to affect bull shark movements, as evidenced in both our study on free-ranging sharks and in reported net captures in both NSW^44^ and South Africa^56^. Conversely, tidal cycle does appear to influence their movements in Sydney Harbour with individuals using areas further from the Harbour entrance at low tide. Although bull sharks are well known for their ability to reside in low salinity waters, these upper regions of the Harbour experience insufficient fresh water input to substantially change salinity levels. It is possible that prey abundance and distribution are the drivers for bull shark occupation of these areas during low tide, as evidenced by the reported movements of potential prey in these regions of Sydney Harbour^50^. Moreover, here we found no influence of total daily rainfall or rainfall from the previous day on the patterns of movement of bull sharks. This is likely due to Sydney Harbour experiencing infrequent, large precipitation events. For example, in Sydney the average monthly rainfall between 1859 and 2010 ranged from a minimum of 69.1 mm in September to a maximum of 130.6 mm in June^57^. This contrasts to seasonal summer and autumn rainfall events experienced in sub-tropical and tropical regions of Australia where rainfall and increased river flow has been shown to alter the patterns of occurrence and movements of bull sharks^31^ and other carcharhinids in nearshore environments^58^.

Movement in large coastal sharks is generally considered to be driven by their desire to exploit seasonally abundant prey^59–61^ or for reproductive requirements^62,63^. While many studies have revealed repeated large-scale movements in response to reproductive philopatry^64^ it is unlikely that directed migrations to Sydney Harbour are associated with reproduction as no sharks have been captured with evidence of recent mating activities and neonates have not been caught in this waterway^18^.

In the case of bull sharks on the east coast of Australia, prey availability has been postulated to influence their abundance in spring on the central Great Barrier Reef where the sharks aggregate to exploit large spawning aggregations of Spanish mackerel (*Scomberomorus commerson*)^21,65^. During summer and autumn, both dolphinfish (*Coryphaena hippurus*) and yellow tail kingfish (*Seriola lalandi*) are abundant in offshore waters of NSW^66^, possibly attracting bull sharks into these waters. Over the period of this study in Sydney Harbour, yellowtail kingfish (*Seriola lalandi*), Australian bonito (*Sarda australis*), frigate mackerel (*Auxis thazard*) and mackerel tuna (*Euthynnus affinis*), among others, were repeatedly observed (pers. obs.), all of which may be potential prey species of bull sharks and may therefore be a driver for their use of this habitat.

The long-term philopatry exhibited by individuals for Sydney Harbour is the first record of this behaviour by bull sharks in temperate waters. Adult bull sharks are known to be highly philopatric to reproductive areas^62,67,68^ and tropical environments^33,69^, yet little was previously known about the fidelity of bull sharks to temperate regions. Over the seven year monitoring period, bull sharks displayed strong inter-annual fidelity to Sydney Harbour with 70% of tagged sharks subsequently returning to the Harbour on one or more years post tagging and 28% of all tagged sharks returning on all possible years. These tagged sharks have often been detected in the central and southern Great Barrier Reef during winter and spring before migrating south to Sydney Harbour in the summer^35^, yet little was known about the abundance and residency of bull sharks in this temperate estuarine environment or the drivers responsible for these patterns of abundance and residency. Here, we found seasonal changes in water temperature to be a key predictor of shark occurrence, with the probability of bull sharks being present in the Harbour increasing when water temperature was ~22°C and generally increasing with increasing temperature. Such seasonal change in water temperature has previously been shown to influence the distribution and abundance of bull sharks, with sharks present in sub-tropical and temperate waters on the east coast of Australia when sea surface temperature was between 20 and 26°C, with peak abundance at 24°C^22^. Similar seasonal patterns of temperature-mediated movements have been found for other elasmobranchs^21,47,70–73^. While temperature appears to be the key driver of the seasonal occurrence of bull sharks in Sydney Harbour, further research is required to determine whether individuals gain physiological advantages for using these seasonally warmer environments as shown for tiger sharks (*Galeocerdo cuvier*)^74^.

Sydney Harbour is Australia’s busiest, most industrialised and urbanised estuary playing a significant economic, social and environmental role for the city of Sydney, housing 5.23 million people^36,75,76^. Understanding and potentially managing shark-human interactions, especially in highly populated estuaries, is important given the growing human population, more people in the water and thus the probability of an unprovoked shark bite occurring being increased^7^. Bull sharks have been implicated in several fatal or severe interactions in New South Wales and around the world^9,17,77^. The need to understand their movement and behaviour from a public safety and risk management perspective is therefore imperative. In this study we highlight that the greatest likelihood of large bull sharks occurring in Sydney Harbour is: (i) when water temperature is around 22°C during the austral summer and autumn and (ii) in areas with water depths less than 5 m and near areas with steeper drop-offs. Bull sharks use shallower water at night, with changes in depth use recorded during crepuscular periods. Yet, whilst we found that bull sharks in Sydney Harbour were not more active during low-light periods (i.e. dawn, dusk and night), we cannot rule out that bull sharks don’t feed during these periods. The higher abundance of bull sharks in Sydney Harbour during the warmer months coincides with greater water-use activities (e.g. swimming, snorkelling, diving, kayaking). Although bull shark encounters are relatively rare, despite regular use by large sharks in high recreational areas in the Harbour, our results support current recommendations for water-users to exercise caution during nocturnal and crepuscular periods to avoid risk of encounters with bull sharks given their sensory adaptations for low-light levels^78^. These recommendations are particularly pertinent when waters are around 22°C and where shallow waters are close to steep drop-offs.

## Methods

### Study location

**Figure 12:**
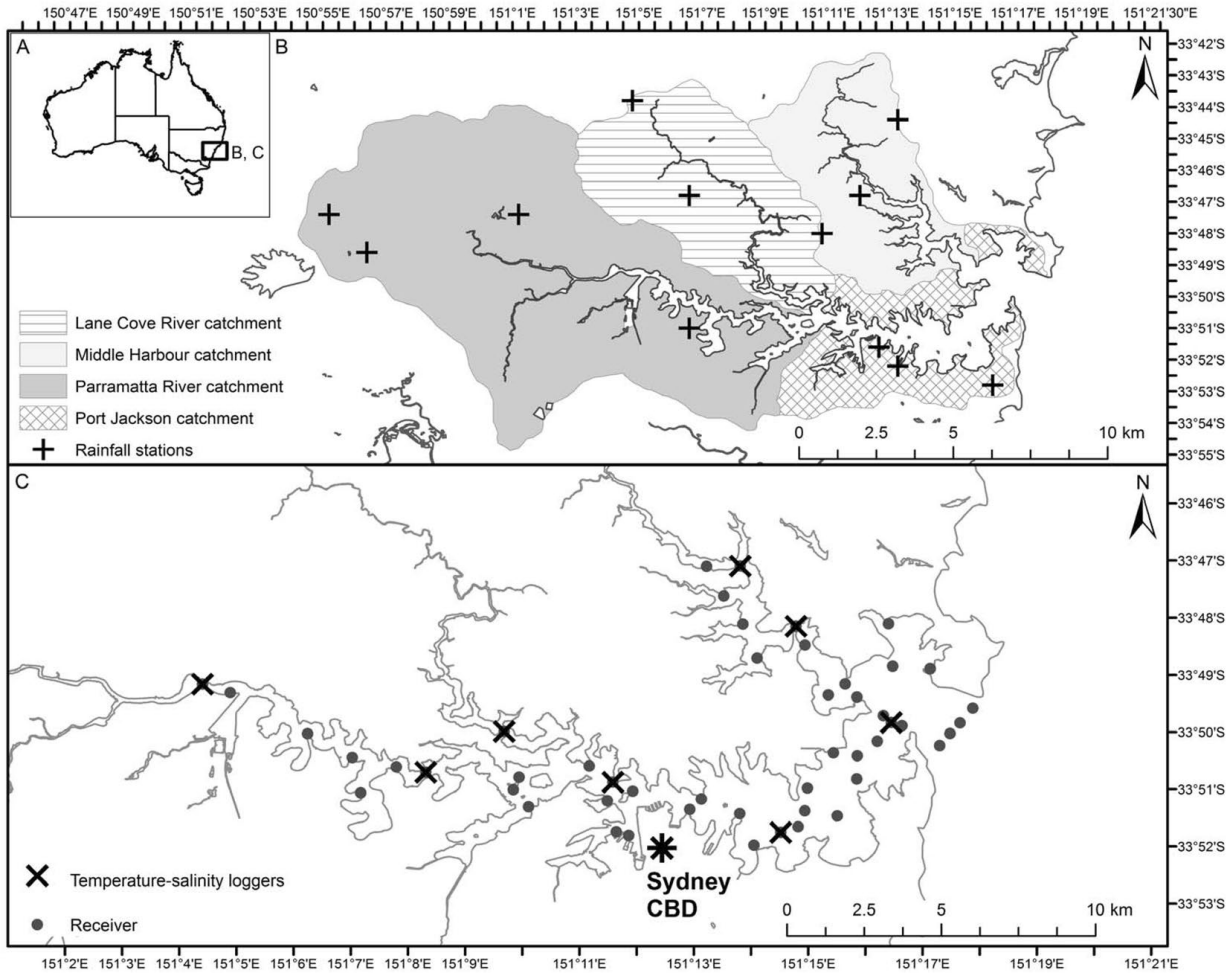
Study location.

Sydney Harbour (~33°51′S, 151°14′E, Fig. 12) is the most urbanised estuary in Australia, surrounded by the city of Sydney which is home to over five million residents and supports a large amount of recreational, commercial and industrial activities^79^. The estuary is a large, deep, drowned river valley approximately 30 km long, 3 km at its widest point and covers an area of 55 km^2^ with numerous tributaries and waterways^80^. The morphology of the seabed is complex and irregular with a series of deep holes up to 47 m deep, however, most embayments are relatively shallow (<15 m). It is fully tidal and has a relatively small freshwater inflow from two rivers, the Parramatta and Lane Cove rivers. Salinity reflects marine conditions but declines after heavy rainfall when there is often a surface layer of fresh water that can extend up to half the length of the Harbour^80^.

### Shark tagging and acoustic array

In 2009, an array of VR2W acoustic receivers (Vemco Ltd, Nova Scotia) was deployed to monitor movements of bull sharks. Receivers were strategically placed from the mouth of the estuary to Parramatta (~30 km upstream, where a man-made weir delimits the main extent of the estuary) at choke points to act as a series of gates throughout the waterway (Fig. 12c). Receivers were deployed at depths between 3 and 8 m and anchored differently depending on the available substratum, with the majority attached to existing navigational structures. Receiver data were downloaded every 6–12 months. Acoustic detections from the four receivers deployed across the Harbour entrance by the Integrated Marine Observing System (IMOS) Animal Tracking Facility (ATF) were accessed via the IMOS AFT database. Sentinel acoustic transmitters were deployed at two locations within Sydney Harbour and indicated that the effective detection range defined as the distance at which detection probability was 50%^81,82^ was approximately 250 m (see Supplementary Material 1).

Between March 2009 and January 2013, shark fishing was done in depths ranging from 1.5 to 29 m using 200 m long bottom-set longlines, consisting of 7 mm braided lead-core rope anchored at each end^18^. Each of these set-lines included 15 snoods spaced approximately 13 m apart. Snoods were 3 m long and made of 3 mm plastic coated stainless steel wire trace with a breaking strain of 400 lb. Each snood was connected to the mainline via a shark clip and had a 16/0 tuna circle hook baited with half a frozen sea mullet (*Mugil cephalus*). A burley canister was attached to the surface floats, at each end of the set-line, with a predefined and consistent frozen mixture made from 500 g of minced Australian pilchard (*Sardinops sagax*), 500 g of chicken layer pellets and 250 ml of tuna oil. The set-lines were deployed two hours before dusk, soaked for two hours then checked, re-baited and soaked for a further two hours before being retrieved. Bottom-set longlines were chosen above other fishing-methods e.g. drum-lines^10^ because they: (i) are known to be successful in catching various species of large sharks targeted commercially in NSW^40^, (ii) provided an increased amount of sampling-effort, and (iii) were least hazardous to other vessels within the heavily congested Harbour waterways.

Captured sharks were brought alongside the research vessel, where they were identified, tail-roped and inverted to induce tonic immobility. The gender, precaudal, fork and total lengths (measured to the nearest cm) of each shark were recorded. Individuals were classified as immature or mature depending on the basis of clasper calcification (males) and published size at maturity of bull sharks reported for males and females 2.2 and 2.3 m, respectively^39^. Captured individuals were tagged with an external identification tag below the first dorsal and surgically implanted with an acoustic transmitter. Of the 40 bull sharks tagged, 23 were tagged with V16TP tags, which reported the depth and temperature of an individual in the water column with every transmission (every 30–90 s) and the rest tagged with V16 tags. Transmitters were programmed on a pseudo-random repeat rate of 30–90 s or 40–80 s and had a battery life of 3260 days. A small (3 cm) incision was made in the ventral midline, the transmitter fitted into the peritoneal cavity and the wound closed using two interrupted sutures. All surgical procedures were done following protocols approved by NSW Department of Primary Industries Fisheries Animal Care and Ethics (07/08) following veterinary training of staff. Sharks were retained for a maximum of 15 minutes during the tagging procedures and the hook removed prior to release.

## Data analysis

### Abundance and residency patterns

Dates of arrival to and departure from Sydney Harbour were calculated for all the tagged sharks. Arrival dates were not available from sharks during the year that they were tagged. No sharks were still present at the end of the study (29 May 2015), enabling the departure dates for each year (since being tagged) to be established for each shark. A seasonal pattern in bull shark occurrence in Sydney Harbour has previously been documented whereby sharks are likely to occur in austral summer and autumn^18^. To enable calculation of annual return and presence in these waters, years were subsequently assessed as commencing in September rather than calendar years. Generalised Linear Mixed Models using Markov Chain Monte Carlo techniques MCMCglmm^83^ were used to test if the month that sharks arrived/departed from the area were influenced by year (to test for inter-annual variation), and size depending on sex (a size-sex interaction). A unique shark identity code was used as a random variable to account for the repeated measures on the same sharks. The maximum number of receivers deployed per year was used as an offset to account for the variation in spatial coverage of the receivers. The MCMCglmm were fitted using the MCMCglmm package^83^ in R^84^. Since month was an ordered categorical variable an ordinal family error distribution was used. Prior to modelling, data exploration was conducted following the general protocol of Zuur *et al*.^85^ using Cleveland dot plots, boxplots, and scatterplots to identify patterns and any outliers.

A generalised additive mixed model (GAMM) was used to determine if there were inter-annual or seasonal variation in the number of days per month that sharks were present in Sydney Harbour. A shark was considered to be present in Sydney Harbour if two or more detections were recorded on the same calendar day (00:00–23:59 local time), to decrease the likelihood of including false detections^86^. Although environmental conditions can influence the detection probability of tags^81,87,88^, a threshold of two detections to indicate a shark being present is well below the maximum possible number (1440 for nominal interval of 60 s) and therefore less likely to be affected by environmental variables^88^. The number of days that sharks were present in the Harbour was calculated for each month and year. This was then divided by either: (i) the number of days in that month, (ii) the number of days since it was tagged, or (iii) the number of days the receivers were deployed, depending on which was the smallest number. This produced a proportion that was ≥ 0 and ≤ 1. Size depending on sex (a size-sex interaction), year (to test for inter-annual variation) and month (to test for seasonal variability) were used as the predictor variables. A cyclic cubic spline was used to model month to account for its cyclic nature. Again, the unique shark identity code was used as a random variable and the number of receivers that were deployed per month/year was used as an offset term. The GAMM was implemented using the *mgcv* package^89,90^ in R with a binomial link function. Inclusion of each of the explanatory variables was assessed using Akaike information criterion for small sample sizes AICc; ‘MuMIn’ package for R^91^. If the difference in AICc of two or more models was less than one, p-values were used to determine if the variables should be included in the model. Model adequacy was checked using standard residual plots, as well as auto-correlation function plots to check for un-modelled temporal correlation. No temporal autocorrelation was evident.

A residency index, defined as the number of days individual sharks were detected in Sydney Harbour divided by the number of days monitored was used to examine shark presence in the Harbour. Residency indices range from 0 to 1, where values close to 1 suggest that sharks spent all their time in the Harbour. To test if residency differed between sexes, a two sample t-test was done. For all metrics, mean values and standard errors (± SE) were calculated.

### Diel, tidal and depth patterns

To test if sharks utilised Sydney Harbour at certain times of the day, a GAMM with a beta distribution was used to model the proportion of detections per shark in each hourly bin. The proportion of detections per hour were divided by the corresponding standardised detection frequency calculated from the sentinel tags^92^ (see Supplementary Material 1). This standardised proportion of detections per shark was used as the response variable and hour of the day as the explanatory variable. Only data from days when the receivers had been deployed the whole day were included so that the number of receivers deployed was the same for each hourly bin. A beta distribution was used as the proportion of detections in each hourly bin was greater than 0 and less than 1. The shark identity code was used as the random effect. Again, the GAMM was modelled using the *mgcv* package in R and model selection was performed as described above.

Similarly, a GAMM was used to test if the sharks used different areas of the Harbour at certain times of the day or tidal states by modelling the distance from the Harbour entrance against hour of the day and tidal height (m). The centre of activity^93^ was calculated for every hourly bin for each shark to give a single measure of distance to the Harbour entrance per hour. Tidal state for Sydney Harbour was obtained using XTide (http://www.flaterco.com/xtide/), provided as the mean estimated height every hour for the period of the array deployment. A cyclic cubic spline was used to model for hour of the day and a thin plate spline used for tide. Again, the shark identity code was used as the random effect and model selection was performed as described above.

To investigate the position in the water column that the sharks were swimming, the depth at which sharks were detected was divided by the maximum depth relative to each corresponding receivers’ effective detection range (250 m radius). The maximum depth within each receiver’s detection range was calculated from a bathymetry map using ArcGIS. This resulted in the position in the water column (with respect to the maximum depth) being expressed as a proportion with values of 1 indicating sharks were swimming along the seafloor and 0 indicating the surface. This was modelled against hour of the day using a binomial GAMM to determine if sharks utilised the water column differently at certain times of the day (see Supplementary Table S1).

### Area use of Sydney Harbour

The total number of detections at each receiver, across the whole study period, was calculated. Since receivers were deployed for varying lengths of time, the total number of detections was divided by the number of days that each receiver was deployed (referred to as ‘the number of detections per day deployed’). A spatial hotspot analysis, the Getis-Ord Gi* hotspot analysis^94^, was implemented in ESRI ArcGIS (version 10.4.1), to identify areas of high use. For a set of weighted features, this analysis determines the correlation of a given data point value (the number of detections per day deployed) with the values in surrounding areas, automatically performing a test of significance (*z*-score) for each area. The resulting Gi* statistics is a z-score that can be any positive or negative value. At a significance level of 0.05, a *z*-score less than −1.96 is classified as a ‘coldspot’ and indicate significantly more intense clustering of very low values. Z-scores greater than 1.96 are classified as a ‘hotspot’ and indicate significant more intense clustering of very high values. Hence, ‘hotspots’ and ‘coldspots’ of shark detections were defined as high (above the mean number of detections per day deployed) and low (below the mean number of detections per day deployed) areas, respectively. The spatial relationship was conceptualised through an inverse distance relationship whereby values closer together are more likely to be correlated and the correlation decreases with distance. To determine if there was inter-annual variability in areas of ‘hotspot’, the same analysis was repeated for each year. To identify if areas of ‘hotspot’ were related to habitat variables, the mean depth and maximum slope within each area of receiver effective detection range (250 m radius) was calculated from a bathymetry map using ArcGIS. A binomial GAMM was used to determine if the mean depth, maximum slope or year were related to whether or not an area was defined as a ‘hotspot’ (i.e. a binary response variable: ‘hotspot’ = 1 or not a ‘hotspot’ = 0). The station name was used as the random effect to account for repeated measures and model selection was conducted using the AICc as described above (see Supplementary Table S2).

### Environmental correlates of shark presence and abundance

Temperature was recorded by Odyssey loggers that were deployed at 12 locations throughout the Harbour from 3^rd^ May 2011 to 26^th^ October 2013 (Fig. 1c). The mean temperature was calculated as the average of all the recordings across the whole of Sydney Harbour for each day the recorders were deployed. Over the same time period, daily cumulative rainfall data from up to three locations within each river catchment were obtained from the Bureau of Meteorology (www.bom.gov.au; Fig. 1b). Moon illumination was obtained from the United States Naval Observatory Astronomical Applications Department (http://aa.usno.navy.mil/data/docs/MoonPhase.php; see Supplementary Table S3).

A two-stage hurdle model was used to test if: (i) the presence-absence of sharks and, (ii) the number of sharks present per day (excluding days when no sharks were present, i.e. abundance > 0) in Sydney Harbour were influenced by mean water temperature, moon illumination, rainfall from that day or rainfall from the previous day. Binomial and Gamma link functions were used to model the presence-absence and abundance of sharks, respectively (see Supplementary Table S4). The number of receivers deployed varied throughout the study and the number of sharks tagged increased as the study progressed. Therefore, an additive offset term with the number of receivers deployed and the number of sharks tagged was used to account for this variability.

## Supplementary information

Supplementary Information

## Data Availability

Data are available from the IMOS Animal Tracking Database (https://animaltracking.aodn.org.au).
