# Supplementary material for: Long-term patterns of abundance, residency and movements of bull sharks (Carcharhinus leucas) in Sydney Harbour, Australia
Source: Sci Rep. 2019 Dec 11;9:18864. doi: 10.1038/s41598-019-54365-x (PMC6906466; doi:10.1038/s41598-019-54365-x)
Supplement: Supplementary file 1 — Supplementary Information [file 41598_2019_54365_MOESM1_ESM.docx]

**Supplementary Material**

**Long-term patterns of abundance, residency and movements of bull sharks (*Carcharhinus leucas*) in Sydney Harbour, Australia**

Amy F. Smoothey^1*^, Kate A. Lee^2^ and Victor M. Peddemors^1^

^1^NSW Department of Primary Industries, Fisheries Research, Sydney Institute of Marine Science, Mosman, NSW 2088, Australia

^2^ Sydney Institute of Marine Science, Mosman, NSW 2088, Australia

*Corresponding author: [amy.smoothey@dpi.nsw.gov.au](mailto:amy.smoothey@dpi.nsw.gov.au)

**Detection probability vs. environmental variables**

**Methods**

VR2AR receivers (with in-built tags) were deployed at two sites (Nielsen Park and Manns Point) in Sydney Harbour between 29^th^ March 2015 and 14^th^ October 2016 in depths between 8-15 m. At Manns Point, VR2ARs were deployed at 150, 300 and 500 m from a VR2W and at Nielsen Park VR2ARs were deployed at 200, 400 and 600 m from VR2W. This allowed assessment of detection range both between the respective VR2Ws and VR2ARs and the VR2ARs themselves (hereafter collectively referred to as “receiver/sentinel tags”) and resulted in distances of 150, 200, 300, 350, 400, 500 and 600 m.

For each receiver/sentinel tag combination, the detection probability was calculated for each hour of deployment by dividing the number of detections by the expected average number of transmissions per hour (40 detections per hour for a nominal/fixed transmission interval of 90 s). The effective detection range, the distance at which the detection probability was 50%^1,2^ was estimated using a generalised additive mixed model (GAMM) with a binomial link function. The detection probability was used as the response variable and distance as the explanatory variable, with the unique receiver ID used as a random effect to account for the lack of independence from data collected by each receiver. Generalised additive mixed models were implemented using the R^3^ “mgcv”^4,5^ package. Model adequacy was checked using standard residual plots, as well as auto-correlation function plots and semi-variogram plots to check for un-modelled spatial and temporal correlation. Acoustic detectability can be affected by the time of day and hinder the interpretation of biological data^6^. To account for the varying detectability throughout the day, standardised detection frequencies (SDFs) were calculated from the sentinel tags^6^.

**Results**

Overall, the distance at which 50% of transmissions were detected was ~250 m. (Fig. S1). The SDFs calculated from the sentinel tags was higher at night than during the day (Fig. S2).

*
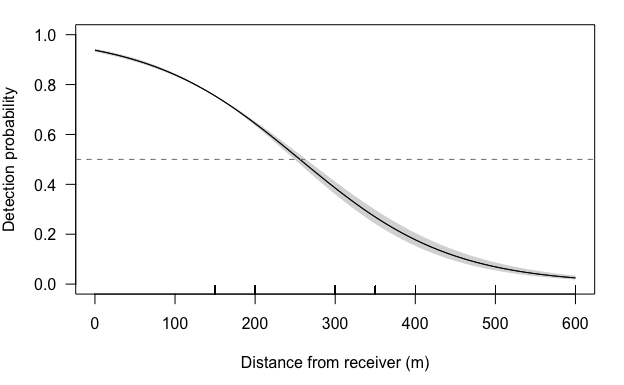
*

Figure S1: Generalised additive mixed model response curve showing the detection probability for sentinel tags deployed at varying distances from receivers. The dashed line represents the effective detection range at which 50% of the transmissions were detected (~250 m).

**
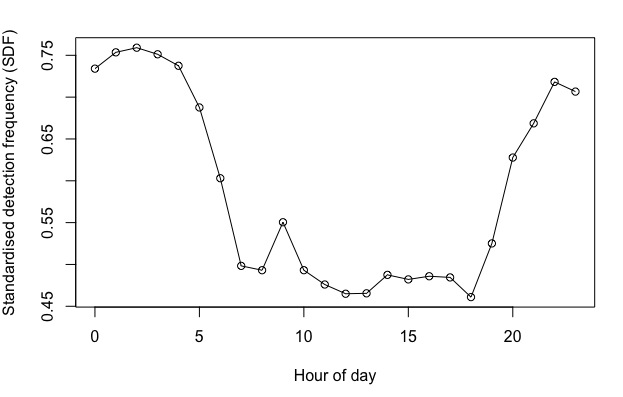
**

Figure S2: Standardised detection frequencies per hourly bin from sentinel tags.


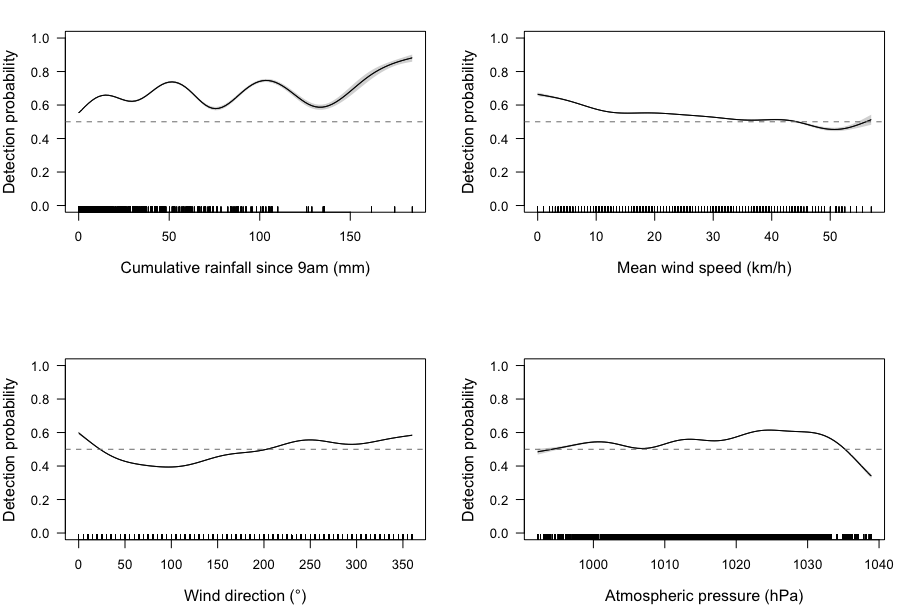


Figure S3: Generalised additive mixed model response curves showing the influence of: a) rainfall, b) wind speed, c) wind direction and d) atmospheric pressure.

Table S1: Generalised additive mixed model results of the influence of hour of the day on the position in the water column that sharks were detected in Sydney Harbour. Random effects included in model. s denotes a smoother term.

| Model | ΔAICc | Deviance explained (%) |
| --- | --- | --- |
| ~ s(hour) | 0 | 20.7 |
| ~ null | 2131.1 | 14.9 |

Table S2: Binomial generalised additive mixed model results of the influence of mean depth, maximum slope and year on the probability of a receiver being deployed within a shark ‘hotspot’. Random effects included in model. s denotes a smoother term.

| Model | ΔAICc | Deviance explained (%) | |
| --- | --- | --- | --- |
| ~ s(mean depth) + s(max slope) + s(year) | 2 | 5.38 |  |
| ~ s(max slope) + s(year) | 3.6 | 2.57 |  |
| ~ s(mean depth) + s(year) | 0.9 | 4.13 |  |
| ~ s(mean depth) + s(max slope) | 0 | 5.28 |  |

Table S3: Daily environmental values for Sydney Harbour. Data for daily cumulative rainfall data were obtained from the Bureau of Meteorology ([www.bom.gov.au](http://www.bom.gov.au)). Moon illumination was obtained from the United States Naval Observatory Astronomical Applications Department (<http://aa.usno.navy.mil/data/docs/MoonPhase.php>).

| Date | Mean temperature (°C) | Total rainfall (mm) | Mean rainfall (mm) | Fraction of moon illumination | Total rainfall  the previous day (mm) |
| --- | --- | --- | --- | --- | --- |
| 3/05/2011 | 19.94 | 29.4 | 1.838 | 0 | 0 |
| 4/05/2011 | 20.03 | 2.8 | 0.175 | 0.01 | 29.4 |
| 5/05/2011 | 20.02 | 0.2 | 0.013 | 0.04 | 2.8 |
| 6/05/2011 | 19.86 | 0 | 0.000 | 0.08 | 0.2 |
| 7/05/2011 | 19.94 | 0 | 0.000 | 0.15 | 0 |
| 8/05/2011 | 19.94 | 0.4 | 0.024 | 0.23 | 0 |
| 9/05/2011 | 19.73 | 0.4 | 0.024 | 0.32 | 0.4 |
| 10/05/2011 | 19.30 | 29.2 | 1.718 | 0.43 | 0.4 |
| 11/05/2011 | 18.93 | 0.4 | 0.024 | 0.54 | 29.2 |
| 12/05/2011 | 18.42 | 0 | 0.000 | 0.65 | 0.4 |
| 13/05/2011 | 18.23 | 0 | 0.000 | 0.76 | 0 |
| 14/05/2011 | 18.01 | 4.7 | 0.276 | 0.85 | 0 |
| 15/05/2011 | 17.74 | 0 | 0.000 | 0.93 | 4.7 |
| 16/05/2011 | 17.63 | 0.2 | 0.012 | 0.98 | 0 |
| 17/05/2011 | 17.60 | 0 | 0.000 | 1 | 0.2 |
| 18/05/2011 | 17.57 | 0.4 | 0.024 | 0.99 | 0 |
| 19/05/2011 | 17.64 | 18 | 1.059 | 0.96 | 0.4 |
| 20/05/2011 | 17.73 | 1.6 | 0.100 | 0.9 | 18 |
| 21/05/2011 | 17.84 | 0.2 | 0.013 | 0.83 | 1.6 |
| 22/05/2011 | 17.90 | 0.2 | 0.013 | 0.75 | 0.2 |
| 23/05/2011 | 17.96 | 19.5 | 1.219 | 0.65 | 0.2 |
| 24/05/2011 | 17.82 | 0 | 0.000 | 0.56 | 19.5 |
| 25/05/2011 | 17.52 | 99.5 | 6.633 | 0.46 | 0 |
| 26/05/2011 | 17.24 | 82 | 5.467 | 0.37 | 99.5 |
| 27/05/2011 | 17.10 | 1.3 | 0.081 | 0.28 | 82 |
| 28/05/2011 | 16.92 | 0.2 | 0.013 | 0.2 | 1.3 |
| 29/05/2011 | 16.85 | 0 | 0.000 | 0.13 | 0.2 |
| 30/05/2011 | 16.78 | 324 | 20.250 | 0.07 | 0 |
| 31/05/2011 | 16.70 | 1042.9 | 61.347 | 0.03 | 324 |
| 1/06/2011 | 16.80 | 145.2 | 9.075 | 0 | 1042.9 |
| 2/06/2011 | 16.90 | 30.3 | 1.894 | 0 | 145.2 |
| 3/06/2011 | 17.01 | 14 | 0.875 | 0.02 | 30.3 |
| 4/06/2011 | 17.11 | 0 | 0.000 | 0.06 | 14 |
| 5/06/2011 | 17.21 | 11.9 | 0.744 | 0.12 | 0 |
| 6/06/2011 | 17.15 | 0 | 0.000 | 0.2 | 11.9 |
| 7/06/2011 | 17.05 | 0 | 0.000 | 0.29 | 0 |
| 8/06/2011 | 16.63 | 0 | 0.000 | 0.4 | 0 |
| 9/06/2011 | 16.27 | 0 | 0.000 | 0.51 | 0 |
| 10/06/2011 | 16.10 | 0 | 0.000 | 0.63 | 0 |
| 11/06/2011 | 16.04 | 63.4 | 4.529 | 0.74 | 0 |
| 12/06/2011 | 15.96 | 195.4 | 13.957 | 0.83 | 63.4 |
| 13/06/2011 | 15.84 | 196.7 | 15.131 | 0.91 | 195.4 |
| 14/06/2011 | 15.84 | 113.7 | 8.121 | 0.97 | 196.7 |
| 15/06/2011 | 15.89 | 238.9 | 15.927 | 0.99 | 113.7 |
| 16/06/2011 | 16.00 | 141.6 | 8.850 | 1 | 238.9 |
| 17/06/2011 | 16.07 | 14.7 | 0.919 | 0.98 | 141.6 |
| 18/06/2011 | 15.90 | 0 | 0.000 | 0.93 | 14.7 |
| 19/06/2011 | 15.95 | 0 | 0.000 | 0.87 | 0 |
| 20/06/2011 | 16.04 | 0 | 0.000 | 0.8 | 0 |
| 21/06/2011 | 15.92 | 0 | 0.000 | 0.71 | 0 |
| 22/06/2011 | 15.57 | 1.8 | 0.113 | 0.62 | 0 |
| 23/06/2011 | 15.43 | 0 | 0.000 | 0.53 | 1.8 |
| 24/06/2011 | 15.54 | 0 | 0.000 | 0.43 | 0 |
| 25/06/2011 | 15.76 | 0 | 0.000 | 0.34 | 0 |
| 26/06/2011 | 15.81 | 0.2 | 0.013 | 0.25 | 0 |
| 27/06/2011 | 15.79 | 0 | 0.000 | 0.17 | 0.2 |
| 28/06/2011 | 15.70 | 1.3 | 0.081 | 0.11 | 0 |
| 29/06/2011 | 15.71 | 65.8 | 4.113 | 0.05 | 1.3 |
| 30/06/2011 | 15.82 | 36.6 | 2.288 | 0.02 | 65.8 |
| 1/07/2011 | 15.92 | 14.9 | 0.993 | 0 | 36.6 |
| 2/07/2011 | 16.04 | 41.1 | 2.569 | 0.01 | 14.9 |
| 3/07/2011 | 16.10 | 0.6 | 0.038 | 0.04 | 41.1 |
| 4/07/2011 | 16.11 | 1 | 0.063 | 0.1 | 0.6 |
| 5/07/2011 | 15.87 | 0.2 | 0.013 | 0.18 | 1 |
| 6/07/2011 | 15.56 | 0 | 0.000 | 0.27 | 0.2 |
| 7/07/2011 | 15.26 | 0 | 0.000 | 0.38 | 0 |
| 8/07/2011 | 14.98 | 0 | 0.000 | 0.49 | 0 |
| 9/07/2011 | 14.86 | 0 | 0.000 | 0.61 | 0 |
| 10/07/2011 | 14.67 | 0 | 0.000 | 0.72 | 0 |
| 11/07/2011 | 14.51 | 0 | 0.000 | 0.81 | 0 |
| 12/07/2011 | 14.46 | 0 | 0.000 | 0.89 | 0 |
| 13/07/2011 | 14.30 | 0 | 0.000 | 0.95 | 0 |
| 14/07/2011 | 14.14 | 0 | 0.000 | 0.99 | 0 |
| 15/07/2011 | 14.03 | 5.1 | 0.340 | 1 | 0 |
| 16/07/2011 | 14.12 | 95.8 | 6.843 | 0.99 | 5.1 |
| 17/07/2011 | 14.33 | 30.1 | 2.007 | 0.96 | 95.8 |
| 18/07/2011 | 14.43 | 7.9 | 0.527 | 0.91 | 30.1 |
| 19/07/2011 | 14.32 | 4.6 | 0.307 | 0.85 | 7.9 |
| 20/07/2011 | 14.26 | 818.9 | 51.181 | 0.77 | 4.6 |
| 21/07/2011 | 14.39 | 401.8 | 25.113 | 0.69 | 818.9 |
| 22/07/2011 | 14.37 | 1274.2 | 79.638 | 0.6 | 401.8 |
| 23/07/2011 | 14.66 | 584.6 | 36.538 | 0.5 | 1274.2 |
| 24/07/2011 | 14.93 | 18.7 | 1.169 | 0.41 | 584.6 |
| 25/07/2011 | 15.13 | 1.2 | 0.075 | 0.31 | 18.7 |
| 26/07/2011 | 15.34 | 11.1 | 0.694 | 0.22 | 1.2 |
| 27/07/2011 | 15.49 | 3.5 | 0.219 | 0.15 | 11.1 |
| 28/07/2011 | 15.56 | 0.4 | 0.025 | 0.08 | 3.5 |
| 29/07/2011 | 15.69 | 0 | 0.000 | 0.03 | 0.4 |
| 30/07/2011 | 15.76 | 0.2 | 0.013 | 0 | 0 |
| 31/07/2011 | 15.86 | 0 | 0.000 | 0 | 0.2 |
| 1/08/2011 | 16.05 | 0 | 0.000 | 0.03 | 0 |
| 2/08/2011 | 16.20 | 0 | 0.000 | 0.08 | 0 |
| 3/08/2011 | 16.31 | 3.6 | 0.225 | 0.16 | 0 |
| 4/08/2011 | 16.39 | 0 | 0.000 | 0.25 | 3.6 |
| 5/08/2011 | 16.51 | 0 | 0.000 | 0.36 | 0 |
| 6/08/2011 | 16.56 | 0 | 0.000 | 0.47 | 0 |
| 7/08/2011 | 16.62 | 11.5 | 0.821 | 0.59 | 0 |
| 8/08/2011 | 16.54 | 57.8 | 3.613 | 0.69 | 11.5 |
| 9/08/2011 | 16.35 | 2.6 | 0.163 | 0.79 | 57.8 |
| 10/08/2011 | 16.15 | 0 | 0.000 | 0.87 | 2.6 |
| 11/08/2011 | 16.00 | 0 | 0.000 | 0.93 | 0 |
| 12/08/2011 | 15.96 | 118.5 | 7.406 | 0.97 | 0 |
| 13/08/2011 | 15.96 | 16.1 | 1.150 | 1 | 118.5 |
| 14/08/2011 | 16.10 | 27.7 | 1.731 | 1 | 16.1 |
| 15/08/2011 | 16.17 | 8.7 | 0.544 | 0.98 | 27.7 |
| 16/08/2011 | 16.27 | 7 | 0.438 | 0.94 | 8.7 |
| 17/08/2011 | 16.25 | 15 | 0.938 | 0.89 | 7 |
| 18/08/2011 | 16.10 | 110.7 | 7.380 | 0.82 | 15 |
| 19/08/2011 | 15.80 | 12.3 | 0.769 | 0.75 | 110.7 |
| 20/08/2011 | 15.78 | 378.2 | 23.638 | 0.66 | 12.3 |
| 21/08/2011 | 15.81 | 22.1 | 1.381 | 0.57 | 378.2 |
| 22/08/2011 | 15.86 | 11.8 | 0.738 | 0.47 | 22.1 |
| 23/08/2011 | 15.97 | 2.7 | 0.169 | 0.37 | 11.8 |
| 24/08/2011 | 16.10 | 5.4 | 0.338 | 0.28 | 2.7 |
| 25/08/2011 | 16.27 | 0.4 | 0.025 | 0.19 | 5.4 |
| 26/08/2011 | 16.50 | 0.2 | 0.013 | 0.11 | 0.4 |
| 27/08/2011 | 16.64 | 2.2 | 0.138 | 0.05 | 0.2 |
| 28/08/2011 | 16.68 | 1.6 | 0.100 | 0.01 | 2.2 |
| 29/08/2011 | 16.84 | 0 | 0.000 | 0 | 1.6 |
| 30/08/2011 | 16.88 | 0.2 | 0.013 | 0.02 | 0 |
| 31/08/2011 | 16.96 | 8.2 | 0.482 | 0.07 | 0.2 |
| 1/09/2011 | 17.14 | 6.7 | 0.394 | 0.14 | 8.2 |
| 2/09/2011 | 17.16 | 0.2 | 0.012 | 0.23 | 6.7 |
| 3/09/2011 | 17.05 | 0 | 0.000 | 0.33 | 0.2 |
| 4/09/2011 | 17.08 | 0 | 0.000 | 0.44 | 0 |
| 5/09/2011 | 17.26 | 0 | 0.000 | 0.55 | 0 |
| 6/09/2011 | 17.40 | 0.2 | 0.012 | 0.66 | 0 |
| 7/09/2011 | 17.40 | 18 | 1.125 | 0.75 | 0.2 |
| 8/09/2011 | 17.30 | 0 | 0.000 | 0.84 | 18 |
| 9/09/2011 | 17.05 | 102.4 | 6.024 | 0.9 | 0 |
| 10/09/2011 | 16.54 | 48.5 | 2.853 | 0.95 | 102.4 |
| 11/09/2011 | 16.33 | 0.7 | 0.041 | 0.99 | 48.5 |
| 12/09/2011 | 16.33 | 1.8 | 0.106 | 1 | 0.7 |
| 13/09/2011 | 16.47 | 0 | 0.000 | 0.99 | 1.8 |
| 14/09/2011 | 16.59 | 1 | 0.059 | 0.97 | 0 |
| 15/09/2011 | 16.87 | 0.8 | 0.047 | 0.93 | 1 |
| 16/09/2011 | 17.04 | 0.2 | 0.012 | 0.87 | 0.8 |
| 17/09/2011 | 17.25 | 0 | 0.000 | 0.8 | 0.2 |
| 18/09/2011 | 17.54 | 0 | 0.000 | 0.72 | 0 |
| 19/09/2011 | 17.83 | 0 | 0.000 | 0.63 | 0 |
| 20/09/2011 | 17.88 | 0 | 0.000 | 0.54 | 0 |
| 21/09/2011 | 17.64 | 0 | 0.000 | 0.44 | 0 |
| 22/09/2011 | 17.71 | 0 | 0.000 | 0.33 | 0 |
| 23/09/2011 | 17.88 | 0 | 0.000 | 0.24 | 0 |
| 24/09/2011 | 17.90 | 1.1 | 0.069 | 0.15 | 0 |
| 25/09/2011 | 17.43 | 408 | 25.500 | 0.08 | 1.1 |
| 26/09/2011 | 17.08 | 505.9 | 31.619 | 0.03 | 408 |
| 27/09/2011 | 17.25 | 107.9 | 6.347 | 0 | 505.9 |
| 28/09/2011 | 17.22 | 0.1 | 0.006 | 0.01 | 107.9 |
| 29/09/2011 | 17.13 | 127.1 | 7.944 | 0.05 | 0.1 |
| 30/09/2011 | 16.83 | 21.1 | 1.319 | 0.11 | 127.1 |
| 1/10/2011 | 16.81 | 0.2 | 0.012 | 0.2 | 21.1 |
| 2/10/2011 | 16.64 | 100.5 | 6.700 | 0.3 | 0.2 |
| 3/10/2011 | 16.61 | 141.3 | 8.312 | 0.4 | 100.5 |
| 4/10/2011 | 16.75 | 6.2 | 0.365 | 0.51 | 141.3 |
| 5/10/2011 | 16.88 | 1.2 | 0.071 | 0.61 | 6.2 |
| 6/10/2011 | 17.04 | 0 | 0.000 | 0.71 | 1.2 |
| 7/10/2011 | 17.24 | 40.6 | 2.388 | 0.79 | 0 |
| 8/10/2011 | 17.51 | 37.1 | 2.319 | 0.87 | 40.6 |
| 9/10/2011 | 17.66 | 43.9 | 2.582 | 0.92 | 37.1 |
| 10/10/2011 | 17.76 | 1.2 | 0.071 | 0.97 | 43.9 |
| 11/10/2011 | 17.84 | 0 | 0.000 | 0.99 | 1.2 |
| 12/10/2011 | 17.96 | 0 | 0.000 | 1 | 0 |
| 13/10/2011 | 18.15 | 3 | 0.176 | 0.99 | 0 |
| 14/10/2011 | 18.02 | 0.5 | 0.029 | 0.96 | 3 |
| 15/10/2011 | 18.17 | 50.4 | 3.150 | 0.92 | 0.5 |
| 16/10/2011 | 18.65 | 2.4 | 0.160 | 0.85 | 50.4 |
| 17/10/2011 | 18.88 | 35 | 2.059 | 0.78 | 2.4 |
| 18/10/2011 | 18.94 | 0.2 | 0.012 | 0.69 | 35 |
| 19/10/2011 | 19.08 | 0 | 0.000 | 0.6 | 0.2 |
| 20/10/2011 | 19.28 | 0 | 0.000 | 0.49 | 0 |
| 21/10/2011 | 19.48 | 0.2 | 0.012 | 0.39 | 0 |
| 22/10/2011 | 19.97 | 0 | 0.000 | 0.28 | 0.2 |
| 23/10/2011 | 20.29 | 0 | 0.000 | 0.19 | 0 |
| 24/10/2011 | 20.43 | 0 | 0.000 | 0.1 | 0 |
| 25/10/2011 | 20.31 | 30.4 | 1.900 | 0.04 | 0 |
| 26/10/2011 | 19.74 | 71.3 | 4.456 | 0.01 | 30.4 |
| 27/10/2011 | 19.39 | 55.3 | 3.456 | 0 | 71.3 |
| 28/10/2011 | 19.37 | 0.2 | 0.012 | 0.03 | 55.3 |
| 29/10/2011 | 19.44 | 0 | 0.000 | 0.08 | 0.2 |
| 30/10/2011 | 19.63 | 0 | 0.000 | 0.16 | 0 |
| 31/10/2011 | 19.84 | 4 | 0.235 | 0.25 | 0 |
| 1/11/2011 | 19.78 | 2.4 | 0.141 | 0.35 | 4 |
| 2/11/2011 | 19.89 | 0.2 | 0.012 | 0.45 | 2.4 |
| 3/11/2011 | 19.98 | 189.7 | 11.856 | 0.55 | 0.2 |
| 4/11/2011 | 20.07 | 63.5 | 3.969 | 0.65 | 189.7 |
| 5/11/2011 | 20.41 | 0.2 | 0.013 | 0.74 | 63.5 |
| 6/11/2011 | 20.45 | 0 | 0.000 | 0.82 | 0.2 |
| 7/11/2011 | 20.79 | 4 | 0.250 | 0.88 | 0 |
| 8/11/2011 | 21.24 | 37.4 | 2.200 | 0.94 | 4 |
| 9/11/2011 | 21.66 | 244.7 | 15.294 | 0.98 | 37.4 |
| 10/11/2011 | 21.67 | 10 | 0.588 | 1 | 244.7 |
| 11/11/2011 | 21.69 | 3.9 | 0.229 | 1 | 10 |
| 12/11/2011 | 21.64 | 1.2 | 0.075 | 0.98 | 3.9 |
| 13/11/2011 | 21.82 | 0 | 0.000 | 0.95 | 1.2 |
| 14/11/2011 | 21.80 | 0 | 0.000 | 0.9 | 0 |
| 15/11/2011 | 21.88 | 0 | 0.000 | 0.83 | 0 |
| 16/11/2011 | 21.64 | 0 | 0.000 | 0.75 | 0 |
| 17/11/2011 | 21.26 | 176.6 | 11.773 | 0.65 | 0 |
| 18/11/2011 | 21.21 | 42.9 | 2.681 | 0.55 | 176.6 |
| 19/11/2011 | 21.41 | 0 | 0.000 | 0.44 | 42.9 |
| 20/11/2011 | 21.60 | 0 | 0.000 | 0.33 | 0 |
| 21/11/2011 | 21.48 | 71 | 4.176 | 0.22 | 0 |
| 22/11/2011 | 20.98 | 83.2 | 5.200 | 0.13 | 71 |
| 23/11/2011 | 20.44 | 544.7 | 34.044 | 0.06 | 83.2 |
| 24/11/2011 | 19.90 | 281.3 | 16.547 | 0.02 | 544.7 |
| 25/11/2011 | 19.75 | 140.2 | 8.247 | 0 | 281.3 |
| 26/11/2011 | 19.77 | 447 | 31.929 | 0.01 | 140.2 |
| 27/11/2011 | 19.92 | 134.4 | 7.906 | 0.05 | 447 |
| 28/11/2011 | 20.21 | 0 | 0.000 | 0.12 | 134.4 |
| 29/11/2011 | 20.44 | 0 | 0.000 | 0.2 | 0 |
| 30/11/2011 | 20.59 | 0 | 0.000 | 0.29 | 0 |
| 1/12/2011 | 20.41 | 43.6 | 2.725 | 0.38 | 0 |
| 2/12/2011 | 20.22 | 30 | 1.875 | 0.48 | 43.6 |
| 3/12/2011 | 20.05 | 0 | 0.000 | 0.58 | 30 |
| 4/12/2011 | 20.00 | 7.8 | 0.459 | 0.67 | 0 |
| 5/12/2011 | 19.58 | 76.7 | 4.794 | 0.76 | 7.8 |
| 6/12/2011 | 19.32 | 66.9 | 3.935 | 0.83 | 76.7 |
| 7/12/2011 | 19.36 | 6.9 | 0.406 | 0.9 | 66.9 |
| 8/12/2011 | 19.55 | 324.9 | 19.112 | 0.95 | 6.9 |
| 9/12/2011 | 19.83 | 56 | 3.294 | 0.98 | 324.9 |
| 10/12/2011 | 20.10 | 2.4 | 0.141 | 1 | 56 |
| 11/12/2011 | 20.29 | 5.1 | 0.300 | 1 | 2.4 |
| 12/12/2011 | 20.32 | 513.9 | 32.119 | 0.97 | 5.1 |
| 13/12/2011 | 20.18 | 95.1 | 5.944 | 0.93 | 513.9 |
| 14/12/2011 | 20.13 | 5 | 0.313 | 0.87 | 95.1 |
| 15/12/2011 | 20.18 | 2.6 | 0.163 | 0.79 | 5 |
| 16/12/2011 | 20.28 | 0 | 0.000 | 0.7 | 2.6 |
| 17/12/2011 | 20.35 | 0.2 | 0.013 | 0.59 | 0 |
| 18/12/2011 | 20.57 | 0 | 0.000 | 0.48 | 0.2 |
| 19/12/2011 | 20.60 | 5.9 | 0.347 | 0.37 | 0 |
| 20/12/2011 | 20.59 | 387.7 | 24.231 | 0.26 | 5.9 |
| 21/12/2011 | 20.76 | 20.7 | 1.218 | 0.17 | 387.7 |
| 22/12/2011 | 20.85 | 8.1 | 0.506 | 0.09 | 20.7 |
| 23/12/2011 | 21.01 | 107.8 | 6.341 | 0.03 | 8.1 |
| 24/12/2011 | 21.30 | 181.2 | 10.659 | 0 | 107.8 |
| 25/12/2011 | 21.69 | 20.5 | 1.206 | 0 | 181.2 |
| 26/12/2011 | 21.78 | 0 | 0.000 | 0.03 | 20.5 |
| 27/12/2011 | 21.83 | 11.5 | 0.676 | 0.08 | 0 |
| 28/12/2011 | 21.83 | 0.4 | 0.024 | 0.14 | 11.5 |
| 29/12/2011 | 22.00 | 0 | 0.000 | 0.22 | 0.4 |
| 30/12/2011 | 22.18 | 10.4 | 0.612 | 0.31 | 0 |
| 31/12/2011 | 22.28 | 9.4 | 0.553 | 0.4 | 10.4 |
| 1/01/2012 | 22.54 | 0.6 | 0.035 | 0.5 | 9.4 |
| 2/01/2012 | 22.98 | 0 | 0.000 | 0.59 | 0.6 |
| 3/01/2012 | 23.27 | 0 | 0.000 | 0.68 | 0 |
| 4/01/2012 | 23.39 | 0 | 0.000 | 0.77 | 0 |
| 5/01/2012 | 23.53 | 0 | 0.000 | 0.84 | 0 |
| 6/01/2012 | 23.04 | 77.5 | 4.844 | 0.9 | 0 |
| 7/01/2012 | 22.84 | 0.5 | 0.031 | 0.95 | 77.5 |
| 8/01/2012 | 22.76 | 0.2 | 0.013 | 0.99 | 0.5 |
| 9/01/2012 | 22.96 | 514.8 | 30.282 | 1 | 0.2 |
| 10/01/2012 | 23.09 | 7.4 | 0.435 | 0.99 | 514.8 |
| 11/01/2012 | 23.04 | 0 | 0.000 | 0.96 | 7.4 |
| 12/01/2012 | 22.52 | 0 | 0.000 | 0.9 | 0 |
| 13/01/2012 | 22.51 | 0 | 0.000 | 0.83 | 0 |
| 14/01/2012 | 22.46 | 53.9 | 3.593 | 0.74 | 0 |
| 15/01/2012 | 22.35 | 403.1 | 25.194 | 0.63 | 53.9 |
| 16/01/2012 | 22.42 | 297.1 | 17.476 | 0.52 | 403.1 |
| 17/01/2012 | 22.63 | 112.4 | 7.493 | 0.41 | 297.1 |
| 18/01/2012 | 22.89 | 5.4 | 0.338 | 0.3 | 112.4 |
| 19/01/2012 | 23.14 | 0.2 | 0.013 | 0.2 | 5.4 |
| 20/01/2012 | 23.35 | 0 | 0.000 | 0.12 | 0.2 |
| 21/01/2012 | 23.34 | 4.9 | 0.327 | 0.05 | 0 |
| 22/01/2012 | 23.32 | 210.2 | 13.138 | 0.02 | 4.9 |
| 23/01/2012 | 23.30 | 6.9 | 0.460 | 0 | 210.2 |
| 24/01/2012 | 23.26 | 10.4 | 0.693 | 0.01 | 6.9 |
| 25/01/2012 | 23.19 | 118.3 | 7.887 | 0.04 | 10.4 |
| 26/01/2012 | 23.13 | 595.8 | 39.720 | 0.09 | 118.3 |
| 27/01/2012 | 23.27 | 66.4 | 4.427 | 0.16 | 595.8 |
| 28/01/2012 | 23.15 | 147.4 | 8.671 | 0.23 | 66.4 |
| 29/01/2012 | 23.23 | 1 | 0.063 | 0.32 | 147.4 |
| 30/01/2012 | 23.18 | 37.7 | 2.356 | 0.41 | 1 |
| 31/01/2012 | 23.04 | 3 | 0.176 | 0.5 | 37.7 |
| 1/02/2012 | 22.87 | 201.7 | 11.865 | 0.6 | 3 |
| 2/02/2012 | 22.33 | 113 | 7.063 | 0.69 | 201.7 |
| 3/02/2012 | 21.72 | 651.7 | 40.731 | 0.78 | 113 |
| 4/02/2012 | 21.31 | 339.9 | 19.994 | 0.85 | 651.7 |
| 5/02/2012 | 21.65 | 4.6 | 0.271 | 0.92 | 339.9 |
| 6/02/2012 | 21.95 | 2 | 0.118 | 0.96 | 4.6 |
| 7/02/2012 | 22.07 | 31.8 | 1.871 | 0.99 | 2 |
| 8/02/2012 | 21.94 | 9.7 | 0.647 | 1 | 31.8 |
| 9/02/2012 | 21.96 | 32.3 | 1.900 | 0.98 | 9.7 |
| 10/02/2012 | 22.02 | 104.4 | 6.141 | 0.93 | 32.3 |
| 11/02/2012 | 22.10 | 23.7 | 1.481 | 0.86 | 104.4 |
| 12/02/2012 | 22.21 | 131.6 | 7.741 | 0.77 | 23.7 |
| 13/02/2012 | 22.39 | 142.3 | 8.371 | 0.67 | 131.6 |
| 14/02/2012 | 22.41 | 33.4 | 1.965 | 0.56 | 142.3 |
| 15/02/2012 | 22.54 | 65.6 | 3.859 | 0.45 | 33.4 |
| 16/02/2012 | 22.81 | 1 | 0.059 | 0.34 | 65.6 |
| 17/02/2012 | 22.78 | 0 | 0.000 | 0.24 | 1 |
| 18/02/2012 | 22.63 | 33.1 | 1.947 | 0.15 | 0 |
| 19/02/2012 | 22.88 | 0 | 0.000 | 0.08 | 33.1 |
| 20/02/2012 | 22.67 | 657.9 | 38.700 | 0.03 | 0 |
| 21/02/2012 | 22.72 | 127 | 7.471 | 0.01 | 657.9 |
| 22/02/2012 | 22.71 | 1.5 | 0.088 | 0 | 127 |
| 23/02/2012 | 22.92 | 0 | 0.000 | 0.02 | 1.5 |
| 24/02/2012 | 23.28 | 1 | 0.059 | 0.05 | 0 |
| 25/02/2012 | 23.37 | 0.6 | 0.035 | 0.1 | 1 |
| 26/02/2012 | 23.07 | 0.9 | 0.053 | 0.17 | 0.6 |
| 27/02/2012 | 23.07 | 0.1 | 0.006 | 0.24 | 0.9 |
| 28/02/2012 | 22.93 | 3.6 | 0.225 | 0.33 | 0.1 |
| 29/02/2012 | 23.06 | 93.2 | 5.825 | 0.42 | 3.6 |
| 1/03/2012 | 22.90 | 459.2 | 27.012 | 0.52 | 93.2 |
| 2/03/2012 | 22.53 | 264.9 | 15.582 | 0.61 | 459.2 |
| 3/03/2012 | 22.30 | 234.1 | 13.771 | 0.71 | 264.9 |
| 4/03/2012 | 22.08 | 48.1 | 2.829 | 0.79 | 234.1 |
| 5/03/2012 | 22.10 | 217.3 | 12.782 | 0.87 | 48.1 |
| 6/03/2012 | 22.28 | 5.8 | 0.341 | 0.94 | 217.3 |
| 7/03/2012 | 21.89 | 2.4 | 0.141 | 0.98 | 5.8 |
| 8/03/2012 | 20.82 | 1252.5 | 78.281 | 1 | 2.4 |
| 9/03/2012 | 20.78 | 203.3 | 12.706 | 0.99 | 1252.5 |
| 10/03/2012 | 21.04 | 9.2 | 0.541 | 0.95 | 203.3 |
| 11/03/2012 | 21.28 | 16 | 0.941 | 0.89 | 9.2 |
| 12/03/2012 | 21.35 | 1 | 0.059 | 0.81 | 16 |
| 13/03/2012 | 21.34 | 20.6 | 1.212 | 0.71 | 1 |
| 14/03/2012 | 21.44 | 0 | 0.000 | 0.6 | 20.6 |
| 15/03/2012 | 21.54 | 0 | 0.000 | 0.48 | 0 |
| 16/03/2012 | 21.60 | 0.2 | 0.012 | 0.38 | 0 |
| 17/03/2012 | 21.69 | 521.1 | 32.569 | 0.28 | 0.2 |
| 18/03/2012 | 21.61 | 63.8 | 3.753 | 0.19 | 521.1 |
| 19/03/2012 | 21.46 | 95.1 | 5.594 | 0.11 | 63.8 |
| 20/03/2012 | 21.31 | 40.2 | 2.513 | 0.06 | 95.1 |
| 21/03/2012 | 21.36 | 7.3 | 0.429 | 0.02 | 40.2 |
| 22/03/2012 | 21.32 | 0.6 | 0.035 | 0 | 7.3 |
| 23/03/2012 | 21.15 | 26.9 | 1.681 | 0 | 0.6 |
| 24/03/2012 | 20.91 | 2 | 0.118 | 0.02 | 26.9 |
| 25/03/2012 | 20.84 | 9.8 | 0.576 | 0.06 | 2 |
| 26/03/2012 | 20.83 | 7.6 | 0.447 | 0.11 | 9.8 |
| 27/03/2012 | 20.91 | 0.8 | 0.047 | 0.18 | 7.6 |
| 28/03/2012 | 20.96 | 27.8 | 1.635 | 0.26 | 0.8 |
| 29/03/2012 | 20.97 | 75.6 | 4.447 | 0.35 | 27.8 |
| 30/03/2012 | 21.17 | 6 | 0.353 | 0.44 | 75.6 |
| 31/03/2012 | 21.53 | 0 | 0.000 | 0.54 | 6 |
| 1/04/2012 | 21.60 | 0 | 0.000 | 0.64 | 0 |
| 2/04/2012 | 21.69 | 30.8 | 1.812 | 0.74 | 0 |
| 3/04/2012 | 21.67 | 0.9 | 0.053 | 0.83 | 30.8 |
| 4/04/2012 | 21.72 | 0 | 0.000 | 0.9 | 0.9 |
| 5/04/2012 | 21.69 | 0 | 0.000 | 0.96 | 0 |
| 6/04/2012 | 21.69 | 0 | 0.000 | 0.99 | 0 |
| 7/04/2012 | 21.57 | 0 | 0.000 | 1 | 0 |
| 8/04/2012 | 21.48 | 0 | 0.000 | 0.97 | 0 |
| 9/04/2012 | 21.21 | 206.5 | 12.147 | 0.92 | 0 |
| 10/04/2012 | 20.73 | 0.2 | 0.012 | 0.84 | 206.5 |
| 11/04/2012 | 20.04 | 0 | 0.000 | 0.74 | 0.2 |
| 12/04/2012 | 19.92 | 52.3 | 3.076 | 0.64 | 0 |
| 13/04/2012 | 20.06 | 0.1 | 0.006 | 0.53 | 52.3 |
| 14/04/2012 | 20.22 | 0 | 0.000 | 0.42 | 0.1 |
| 15/04/2012 | 20.42 | 0 | 0.000 | 0.32 | 0 |
| 16/04/2012 | 20.52 | 0 | 0.000 | 0.23 | 0 |
| 17/04/2012 | 20.48 | 124.6 | 7.329 | 0.15 | 0 |
| 18/04/2012 | 20.12 | 957.6 | 56.329 | 0.09 | 124.6 |
| 19/04/2012 | 20.04 | 1255.4 | 73.847 | 0.04 | 957.6 |
| 20/04/2012 | 20.21 | 26.7 | 1.571 | 0.01 | 1255.4 |
| 21/04/2012 | 20.38 | 0.7 | 0.041 | 0 | 26.7 |
| 22/04/2012 | 20.47 | 0.8 | 0.047 | 0.01 | 0.7 |
| 23/04/2012 | 20.52 | 15 | 0.882 | 0.03 | 0.8 |
| 24/04/2012 | 20.50 | 48.6 | 2.859 | 0.07 | 15 |
| 31/10/2012 | 18.91 | 0 | 0.000 | 0.98 | 48.6 |
| 1/11/2012 | 18.61 | 1 | 0.063 | 0.95 | 0 |
| 2/11/2012 | 18.29 | 0 | 0.000 | 0.9 | 1 |
| 3/11/2012 | 18.30 | 37.9 | 2.369 | 0.84 | 0 |
| 4/11/2012 | 18.47 | 17.6 | 1.100 | 0.76 | 37.9 |
| 5/11/2012 | 18.83 | 1.4 | 0.088 | 0.68 | 17.6 |
| 6/11/2012 | 19.24 | 2 | 0.125 | 0.58 | 1.4 |
| 7/11/2012 | 19.24 | 0.4 | 0.025 | 0.48 | 2 |
| 8/11/2012 | 19.00 | 18.2 | 1.138 | 0.38 | 0.4 |
| 9/11/2012 | 18.83 | 163.8 | 10.238 | 0.28 | 18.2 |
| 10/11/2012 | 18.73 | 50 | 3.125 | 0.19 | 163.8 |
| 11/11/2012 | 18.80 | 0.4 | 0.025 | 0.11 | 50 |
| 12/11/2012 | 18.92 | 0 | 0.000 | 0.04 | 0.4 |
| 13/11/2012 | 18.65 | 0 | 0.000 | 0.01 | 0 |
| 14/11/2012 | 18.43 | 15.6 | 0.975 | 0 | 0 |
| 15/11/2012 | 18.59 | 4.4 | 0.275 | 0.03 | 15.6 |
| 16/11/2012 | 18.50 | 23.8 | 1.983 | 0.08 | 4.4 |
| 17/11/2012 | 18.36 | 161.7 | 10.106 | 0.15 | 23.8 |
| 18/11/2012 | 18.67 | 0.2 | 0.013 | 0.25 | 161.7 |
| 19/11/2012 | 18.50 | 0 | 0.000 | 0.35 | 0.2 |
| 20/11/2012 | 18.48 | 56.8 | 3.550 | 0.46 | 0 |
| 21/11/2012 | 18.72 | 3.8 | 0.238 | 0.56 | 56.8 |
| 22/11/2012 | 18.76 | 0.6 | 0.038 | 0.66 | 3.8 |
| 23/11/2012 | 18.83 | 0 | 0.000 | 0.76 | 0.6 |
| 24/11/2012 | 19.15 | 0 | 0.000 | 0.83 | 0 |
| 25/11/2012 | 19.56 | 0 | 0.000 | 0.9 | 0 |
| 26/11/2012 | 19.45 | 0 | 0.000 | 0.95 | 0 |
| 27/11/2012 | 19.59 | 17.1 | 1.315 | 0.98 | 0 |
| 28/11/2012 | 19.68 | 163.5 | 10.900 | 1 | 17.1 |
| 29/11/2012 | 20.05 | 12.2 | 0.813 | 1 | 163.5 |
| 30/11/2012 | 20.61 | 11.7 | 0.731 | 0.98 | 12.2 |
| 1/12/2012 | 20.57 | 26.8 | 1.787 | 0.94 | 11.7 |
| 2/12/2012 | 19.97 | 4.6 | 0.288 | 0.89 | 26.8 |
| 3/12/2012 | 20.09 | 30.3 | 1.894 | 0.82 | 4.6 |
| 4/12/2012 | 20.15 | 16.3 | 1.087 | 0.74 | 30.3 |
| 5/12/2012 | 20.08 | 0 | 0.000 | 0.65 | 16.3 |
| 6/12/2012 | 20.01 | 0 | 0.000 | 0.55 | 0 |
| 7/12/2012 | 20.08 | 0 | 0.000 | 0.44 | 0 |
| 8/12/2012 | 20.37 | 0 | 0.000 | 0.34 | 0 |
| 9/12/2012 | 20.35 | 0 | 0.000 | 0.23 | 0 |
| 10/12/2012 | 19.80 | 33.3 | 2.081 | 0.14 | 0 |
| 11/12/2012 | 19.54 | 10.8 | 0.720 | 0.07 | 33.3 |
| 12/12/2012 | 19.69 | 45.2 | 3.013 | 0.02 | 10.8 |
| 13/12/2012 | 20.03 | 2.9 | 0.193 | 0.01 | 45.2 |
| 14/12/2012 | 20.31 | 0 | 0.000 | 0 | 2.9 |
| 15/12/2012 | 20.22 | 0 | 0.000 | 0.02 | 0 |
| 16/12/2012 | 20.09 | 1 | 0.067 | 0.07 | 0 |
| 17/12/2012 | 19.79 | 8.9 | 0.593 | 0.14 | 1 |
| 18/12/2012 | 19.78 | 0.2 | 0.013 | 0.23 | 8.9 |
| 19/12/2012 | 20.32 | 0.5 | 0.033 | 0.33 | 0.2 |
| 20/12/2012 | 20.23 | 0 | 0.000 | 0.43 | 0.5 |
| 21/12/2012 | 19.96 | 0 | 0.000 | 0.53 | 0 |
| 22/12/2012 | 20.28 | 0.4 | 0.027 | 0.63 | 0 |
| 23/12/2012 | 20.93 | 0 | 0.000 | 0.72 | 0.4 |
| 24/12/2012 | 21.39 | 0.2 | 0.013 | 0.8 | 0 |
| 25/12/2012 | 20.35 | 119.2 | 7.947 | 0.87 | 0.2 |
| 26/12/2012 | 20.04 | 335 | 22.333 | 0.93 | 119.2 |
| 27/12/2012 | 20.20 | 0 | 0.000 | 0.97 | 335 |
| 28/12/2012 | 20.37 | 0.2 | 0.013 | 0.99 | 0 |
| 29/12/2012 | 20.39 | 0 | 0.000 | 1 | 0.2 |
| 30/12/2012 | 20.60 | 3 | 0.200 | 0.99 | 0 |
| 31/12/2012 | 21.01 | 2.2 | 0.147 | 0.96 | 3 |
| 1/01/2013 | 21.07 | 0 | 0.000 | 0.91 | 2.2 |
| 2/01/2013 | 20.95 | 0 | 0.000 | 0.84 | 0 |
| 3/01/2013 | 20.97 | 0.2 | 0.013 | 0.76 | 0 |
| 4/01/2013 | 21.59 | 0 | 0.000 | 0.67 | 0.2 |
| 5/01/2013 | 21.86 | 0 | 0.000 | 0.57 | 0 |
| 6/01/2013 | 21.80 | 0 | 0.000 | 0.46 | 0 |
| 7/01/2013 | 21.63 | 0 | 0.000 | 0.35 | 0 |
| 8/01/2013 | 22.07 | 0 | 0.000 | 0.24 | 0 |
| 9/01/2013 | 21.58 | 0 | 0.000 | 0.15 | 0 |
| 10/01/2013 | 21.60 | 0 | 0.000 | 0.08 | 0 |
| 11/01/2013 | 21.69 | 0 | 0.000 | 0.03 | 0 |
| 12/01/2013 | 21.49 | 0 | 0.000 | 0 | 0 |
| 13/01/2013 | 21.28 | 0.2 | 0.013 | 0.01 | 0 |
| 14/01/2013 | 21.25 | 189.3 | 11.831 | 0.04 | 0.2 |
| 15/01/2013 | 21.28 | 3.3 | 0.206 | 0.1 | 189.3 |
| 16/01/2013 | 21.58 | 0 | 0.000 | 0.18 | 3.3 |
| 17/01/2013 | 21.83 | 0 | 0.000 | 0.26 | 0 |
| 18/01/2013 | 22.03 | 0 | 0.000 | 0.36 | 0 |
| 19/01/2013 | 21.90 | 9.9 | 0.660 | 0.46 | 0 |
| 20/01/2013 | 21.81 | 13.6 | 0.907 | 0.55 | 9.9 |
| 21/01/2013 | 22.01 | 5.3 | 0.331 | 0.65 | 13.6 |
| 22/01/2013 | 22.35 | 0.6 | 0.040 | 0.73 | 5.3 |
| 23/01/2013 | 21.85 | 6.2 | 0.413 | 0.81 | 0.6 |
| 24/01/2013 | 21.78 | 2 | 0.133 | 0.88 | 6.2 |
| 25/01/2013 | 22.35 | 2 | 0.125 | 0.93 | 2 |
| 26/01/2013 | 22.78 | 0 | 0.000 | 0.97 | 2 |
| 27/01/2013 | 22.64 | 265.2 | 16.575 | 0.99 | 0 |
| 28/01/2013 | 22.56 | 229.8 | 14.363 | 1 | 265.2 |
| 29/01/2013 | 22.10 | 1931.8 | 113.635 | 0.98 | 229.8 |
| 30/01/2013 | 21.21 | 6.3 | 0.394 | 0.94 | 1931.8 |
| 31/01/2013 | 21.26 | 1.6 | 0.100 | 0.89 | 6.3 |
| 1/02/2013 | 21.14 | 0.5 | 0.031 | 0.81 | 1.6 |
| 2/02/2013 | 20.81 | 809.1 | 47.594 | 0.72 | 0.5 |
| 3/02/2013 | 20.70 | 31.4 | 2.093 | 0.62 | 809.1 |
| 4/02/2013 | 20.61 | 20 | 1.250 | 0.51 | 31.4 |
| 5/02/2013 | 20.70 | 3.2 | 0.188 | 0.39 | 20 |
| 6/02/2013 | 20.86 | 5 | 0.294 | 0.28 | 3.2 |
| 7/02/2013 | 21.42 | 0 | 0.000 | 0.19 | 5 |
| 8/02/2013 | 21.83 | 0 | 0.000 | 0.1 | 0 |
| 9/02/2013 | 21.96 | 0 | 0.000 | 0.04 | 0 |
| 10/02/2013 | 21.40 | 0 | 0.000 | 0.01 | 0 |
| 11/02/2013 | 21.28 | 164.5 | 9.676 | 0 | 0 |
| 12/02/2013 | 21.29 | 152.6 | 8.976 | 0.02 | 164.5 |
| 13/02/2013 | 21.15 | 23 | 1.353 | 0.06 | 152.6 |
| 14/02/2013 | 21.19 | 1.8 | 0.113 | 0.12 | 23 |
| 15/02/2013 | 21.28 | 29 | 1.706 | 0.2 | 1.8 |
| 16/02/2013 | 21.22 | 28.8 | 1.920 | 0.29 | 29 |
| 17/02/2013 | 21.41 | 142.4 | 9.493 | 0.38 | 28.8 |
| 18/02/2013 | 21.59 | 10.6 | 0.624 | 0.47 | 142.4 |
| 19/02/2013 | 21.72 | 9.7 | 0.571 | 0.57 | 10.6 |
| 20/02/2013 | 21.75 | 11.5 | 0.676 | 0.66 | 9.7 |
| 21/02/2013 | 22.09 | 24.6 | 1.447 | 0.74 | 11.5 |
| 22/02/2013 | 22.30 | 15 | 0.882 | 0.82 | 24.6 |
| 23/02/2013 | 22.43 | 312.5 | 22.321 | 0.89 | 15 |
| 24/02/2013 | 22.53 | 662.3 | 47.307 | 0.94 | 312.5 |
| 25/02/2013 | 22.75 | 84.2 | 5.263 | 0.98 | 662.3 |
| 26/02/2013 | 22.69 | 0.7 | 0.044 | 1 | 84.2 |
| 27/02/2013 | 22.10 | 0 | 0.000 | 0.99 | 0.7 |
| 28/02/2013 | 20.38 | 0 | 0.000 | 0.97 | 0 |
| 1/03/2013 | 19.70 | 576.5 | 33.912 | 0.92 | 0 |
| 2/03/2013 | 19.85 | 326.1 | 19.182 | 0.85 | 576.5 |
| 3/03/2013 | 20.05 | 112 | 7.000 | 0.76 | 326.1 |
| 4/03/2013 | 20.38 | 92.3 | 5.769 | 0.65 | 112 |
| 5/03/2013 | 20.58 | 4.4 | 0.275 | 0.54 | 92.3 |
| 6/03/2013 | 20.73 | 0.3 | 0.019 | 0.43 | 4.4 |
| 7/03/2013 | 20.96 | 0.2 | 0.013 | 0.32 | 0.3 |
| 8/03/2013 | 21.50 | 0.5 | 0.029 | 0.22 | 0.2 |
| 9/03/2013 | 21.96 | 1 | 0.063 | 0.13 | 0.5 |
| 10/03/2013 | 22.04 | 0 | 0.000 | 0.07 | 1 |
| 11/03/2013 | 22.18 | 0.2 | 0.013 | 0.02 | 0 |
| 12/03/2013 | 22.40 | 2.7 | 0.169 | 0 | 0.2 |
| 13/03/2013 | 22.58 | 2.8 | 0.175 | 0.01 | 2.7 |
| 14/03/2013 | 22.16 | 0 | 0.000 | 0.03 | 2.8 |
| 15/03/2013 | 21.18 | 33.4 | 2.088 | 0.08 | 0 |
| 16/03/2013 | 21.70 | 8.6 | 0.573 | 0.14 | 33.4 |
| 17/03/2013 | 21.63 | 6.2 | 0.388 | 0.22 | 8.6 |
| 18/03/2013 | 21.50 | 0.2 | 0.013 | 0.3 | 6.2 |
| 19/03/2013 | 21.53 | 1.6 | 0.100 | 0.39 | 0.2 |
| 20/03/2013 | 21.65 | 1.9 | 0.119 | 0.48 | 1.6 |
| 21/03/2013 | 21.68 | 0.4 | 0.025 | 0.58 | 1.9 |
| 22/03/2013 | 21.69 | 0 | 0.000 | 0.67 | 0.4 |
| 23/03/2013 | 19.92 | 21 | 1.313 | 0.76 | 0 |
| 24/03/2013 | 19.23 | 13.4 | 0.838 | 0.84 | 21 |
| 25/03/2013 | 19.66 | 1 | 0.063 | 0.91 | 13.4 |
| 26/03/2013 | 20.97 | 1.8 | 0.106 | 0.96 | 1 |
| 27/03/2013 | 21.19 | 0 | 0.000 | 0.99 | 1.8 |
| 28/03/2013 | 21.25 | 0 | 0.000 | 1 | 0 |
| 29/03/2013 | 20.62 | 54.1 | 3.864 | 0.98 | 0 |
| 30/03/2013 | 20.81 | 4 | 0.267 | 0.94 | 54.1 |
| 31/03/2013 | 21.00 | 3.8 | 0.238 | 0.87 | 4 |
| 1/04/2013 | 21.04 | 57.9 | 3.406 | 0.79 | 3.8 |
| 2/04/2013 | 21.71 | 2.4 | 0.150 | 0.69 | 57.9 |
| 3/04/2013 | 21.81 | 239.8 | 14.988 | 0.58 | 2.4 |
| 4/04/2013 | 21.66 | 623.9 | 36.700 | 0.46 | 239.8 |
| 5/04/2013 | 21.79 | 171.7 | 10.731 | 0.35 | 623.9 |
| 6/04/2013 | 21.73 | 32.3 | 2.019 | 0.25 | 171.7 |
| 7/04/2013 | 21.78 | 42.4 | 2.494 | 0.16 | 32.3 |
| 8/04/2013 | 21.79 | 4.8 | 0.300 | 0.09 | 42.4 |
| 9/04/2013 | 21.80 | 27.7 | 1.629 | 0.04 | 4.8 |
| 10/04/2013 | 21.72 | 33.2 | 1.953 | 0.01 | 27.7 |
| 11/04/2013 | 21.69 | 0 | 0.000 | 0 | 33.2 |
| 12/04/2013 | 21.69 | 0 | 0.000 | 0.01 | 0 |
| 13/04/2013 | 21.63 | 0 | 0.000 | 0.05 | 0 |
| 14/04/2013 | 21.35 | 0 | 0.000 | 0.09 | 0 |
| 15/04/2013 | 20.96 | 0 | 0.000 | 0.16 | 0 |
| 16/04/2013 | 20.68 | 156.7 | 10.447 | 0.23 | 0 |
| 17/04/2013 | 20.87 | 30.9 | 1.931 | 0.32 | 156.7 |
| 18/04/2013 | 20.96 | 27.2 | 1.700 | 0.41 | 30.9 |
| 19/04/2013 | 20.73 | 19.6 | 1.225 | 0.5 | 27.2 |
| 20/04/2013 | 20.23 | 344.2 | 22.947 | 0.6 | 19.6 |
| 21/04/2013 | 19.96 | 411.5 | 25.719 | 0.7 | 344.2 |
| 22/04/2013 | 19.95 | 18.6 | 1.094 | 0.79 | 411.5 |
| 23/04/2013 | 19.96 | 0.2 | 0.013 | 0.87 | 18.6 |
| 24/04/2013 | 19.90 | 0 | 0.000 | 0.93 | 0.2 |
| 25/04/2013 | 19.83 | 0 | 0.000 | 0.98 | 0 |
| 26/04/2013 | 19.78 | 0 | 0.000 | 1 | 0 |
| 27/04/2013 | 19.78 | 0.2 | 0.013 | 0.99 | 0 |
| 28/04/2013 | 19.82 | 0 | 0.000 | 0.96 | 0.2 |
| 29/04/2013 | 19.87 | 4.4 | 0.275 | 0.9 | 0 |
| 30/04/2013 | 19.95 | 0 | 0.000 | 0.81 | 4.4 |
| 1/05/2013 | 20.03 | 0.2 | 0.013 | 0.71 | 0 |
| 2/05/2013 | 19.98 | 0.2 | 0.013 | 0.61 | 0.2 |
| 3/05/2013 | 19.75 | 0.8 | 0.050 | 0.49 | 0.2 |
| 4/05/2013 | 19.59 | 0 | 0.000 | 0.38 | 0.8 |
| 5/05/2013 | 19.46 | 0 | 0.000 | 0.28 | 0 |
| 6/05/2013 | 19.34 | 0 | 0.000 | 0.19 | 0 |
| 7/05/2013 | 19.23 | 0 | 0.000 | 0.12 | 0 |
| 8/05/2013 | 19.20 | 7.4 | 0.463 | 0.06 | 0 |
| 9/05/2013 | 19.20 | 0 | 0.000 | 0.02 | 7.4 |
| 10/05/2013 | 19.19 | 0.2 | 0.013 | 0 | 0 |
| 11/05/2013 | 19.17 | 0.2 | 0.013 | 0 | 0.2 |
| 12/05/2013 | 19.16 | 2.2 | 0.138 | 0.02 | 0.2 |
| 13/05/2013 | 19.12 | 0.2 | 0.013 | 0.06 | 2.2 |
| 14/05/2013 | 19.08 | 8.4 | 0.525 | 0.11 | 0.2 |
| 15/05/2013 | 18.85 | 1.9 | 0.127 | 0.18 | 8.4 |
| 16/05/2013 | 18.55 | 0 | 0.000 | 0.25 | 1.9 |
| 17/05/2013 | 18.28 | 0 | 0.000 | 0.34 | 0 |
| 18/05/2013 | 18.02 | 0 | 0.000 | 0.44 | 0 |
| 19/05/2013 | 17.76 | 0 | 0.000 | 0.54 | 0 |
| 20/05/2013 | 17.53 | 0 | 0.000 | 0.64 | 0 |
| 21/05/2013 | 17.41 | 0 | 0.000 | 0.74 | 0 |
| 22/05/2013 | 17.35 | 0 | 0.000 | 0.83 | 0 |
| 23/05/2013 | 17.57 | 375.9 | 25.060 | 0.9 | 0 |
| 24/05/2013 | 17.60 | 327.4 | 23.386 | 0.96 | 375.9 |
| 25/05/2013 | 17.82 | 200.1 | 12.506 | 0.99 | 327.4 |
| 26/05/2013 | 17.99 | 0.7 | 0.047 | 1 | 200.1 |
| 27/05/2013 | 18.01 | 17.6 | 1.173 | 0.97 | 0.7 |
| 28/05/2013 | 18.23 | 178.7 | 12.764 | 0.92 | 17.6 |
| 29/05/2013 | 18.23 | 21.7 | 1.356 | 0.84 | 178.7 |
| 30/05/2013 | 18.08 | 0.2 | 0.013 | 0.74 | 21.7 |
| 31/05/2013 | 18.15 | 2.2 | 0.138 | 0.64 | 0.2 |
| 1/06/2013 | 18.04 | 0.4 | 0.024 | 0.53 | 2.2 |
| 2/06/2013 | 18.06 | 249.9 | 15.619 | 0.42 | 0.4 |
| 3/06/2013 | 17.89 | 367.9 | 22.994 | 0.32 | 249.9 |
| 4/06/2013 | 17.75 | 0.1 | 0.007 | 0.23 | 367.9 |
| 5/06/2013 | 17.60 | 1.4 | 0.088 | 0.15 | 0.1 |
| 6/06/2013 | 17.65 | 30 | 1.875 | 0.09 | 1.4 |
| 7/06/2013 | 17.73 | 1.6 | 0.100 | 0.04 | 30 |
| 8/06/2013 | 17.88 | 2.5 | 0.147 | 0.01 | 1.6 |
| 9/06/2013 | 17.78 | 0 | 0.000 | 0 | 2.5 |
| 10/06/2013 | 17.83 | 0.2 | 0.013 | 0.01 | 0 |
| 11/06/2013 | 17.85 | 22.3 | 1.312 | 0.03 | 0.2 |
| 12/06/2013 | 17.82 | 2.3 | 0.144 | 0.07 | 22.3 |
| 13/06/2013 | 17.67 | 119.6 | 7.035 | 0.13 | 2.3 |
| 14/06/2013 | 17.53 | 0.3 | 0.019 | 0.2 | 119.6 |
| 15/06/2013 | 17.44 | 1.5 | 0.088 | 0.29 | 0.3 |
| 16/06/2013 | 17.18 | 0.7 | 0.044 | 0.38 | 1.5 |
| 17/06/2013 | 16.94 | 0 | 0.000 | 0.48 | 0.7 |
| 18/06/2013 | 16.66 | 0 | 0.000 | 0.59 | 0 |
| 19/06/2013 | 16.56 | 140.8 | 8.800 | 0.69 | 0 |
| 20/06/2013 | 16.36 | 23.8 | 1.400 | 0.79 | 140.8 |
| 21/06/2013 | 16.23 | 0.8 | 0.047 | 0.88 | 23.8 |
| 22/06/2013 | 16.12 | 26.9 | 1.582 | 0.94 | 0.8 |
| 23/06/2013 | 16.03 | 585.3 | 34.429 | 0.99 | 26.9 |
| 24/06/2013 | 15.97 | 842.7 | 49.571 | 1 | 585.3 |
| 25/06/2013 | 16.03 | 155.7 | 9.159 | 0.98 | 842.7 |
| 26/06/2013 | 16.05 | 332.3 | 19.547 | 0.93 | 155.7 |
| 27/06/2013 | 16.15 | 60.6 | 3.788 | 0.86 | 332.3 |
| 28/06/2013 | 16.23 | 133.9 | 8.369 | 0.78 | 60.6 |
| 29/06/2013 | 16.38 | 413 | 25.813 | 0.68 | 133.9 |
| 30/06/2013 | 16.60 | 595.9 | 35.053 | 0.57 | 413 |
| 1/07/2013 | 16.69 | 74.9 | 4.406 | 0.47 | 595.9 |
| 2/07/2013 | 17.16 | 0.4 | 0.025 | 0.37 | 74.9 |
| 3/07/2013 | 17.39 | 0.7 | 0.044 | 0.27 | 0.4 |
| 4/07/2013 | 17.48 | 0.2 | 0.013 | 0.19 | 0.7 |
| 5/07/2013 | 17.72 | 0 | 0.000 | 0.12 | 0.2 |
| 6/07/2013 | 17.71 | 0.2 | 0.013 | 0.07 | 0 |
| 7/07/2013 | 17.50 | 0 | 0.000 | 0.03 | 0.2 |
| 8/07/2013 | 17.25 | 0 | 0.000 | 0.01 | 0 |
| 9/07/2013 | 17.03 | 8.2 | 0.482 | 0 | 0 |
| 10/07/2013 | 16.83 | 68.2 | 4.012 | 0.02 | 8.2 |
| 11/07/2013 | 16.70 | 3.2 | 0.188 | 0.05 | 68.2 |
| 12/07/2013 | 16.68 | 1 | 0.059 | 0.1 | 3.2 |
| 13/07/2013 | 16.62 | 0.4 | 0.025 | 0.16 | 1 |
| 14/07/2013 | 16.61 | 1 | 0.067 | 0.24 | 0.4 |
| 15/07/2013 | 16.53 | 8.1 | 0.476 | 0.34 | 1 |
| 16/07/2013 | 16.65 | 12.8 | 0.800 | 0.44 | 8.1 |
| 17/07/2013 | 16.71 | 81.4 | 4.788 | 0.55 | 12.8 |
| 18/07/2013 | 16.77 | 0 | 0.000 | 0.65 | 81.4 |
| 19/07/2013 | 16.69 | 0 | 0.000 | 0.76 | 0 |
| 20/07/2013 | 16.77 | 25.7 | 1.512 | 0.85 | 0 |
| 21/07/2013 | 16.53 | 0.6 | 0.038 | 0.93 | 25.7 |
| 22/07/2013 | 16.07 | 0 | 0.000 | 0.98 | 0.6 |
| 23/07/2013 | 15.68 | 0 | 0.000 | 1 | 0 |
| 24/07/2013 | 15.40 | 0 | 0.000 | 0.99 | 0 |
| 25/07/2013 | 15.30 | 0 | 0.000 | 0.95 | 0 |
| 26/07/2013 | 15.33 | 0 | 0.000 | 0.89 | 0 |
| 27/07/2013 | 15.34 | 0 | 0.000 | 0.81 | 0 |
| 28/07/2013 | 15.32 | 0 | 0.000 | 0.72 | 0 |
| 29/07/2013 | 15.21 | 0 | 0.000 | 0.62 | 0 |
| 30/07/2013 | 15.29 | 0 | 0.000 | 0.52 | 0 |
| 31/07/2013 | 15.45 | 95 | 5.588 | 0.42 | 0 |
| 1/08/2013 | 15.34 | 0.6 | 0.038 | 0.33 | 95 |
| 2/08/2013 | 15.30 | 0 | 0.000 | 0.24 | 0.6 |
| 3/08/2013 | 15.25 | 0 | 0.000 | 0.17 | 0 |
| 4/08/2013 | 15.22 | 0 | 0.000 | 0.1 | 0 |
| 5/08/2013 | 15.22 | 0 | 0.000 | 0.05 | 0 |
| 6/08/2013 | 15.14 | 0.5 | 0.031 | 0.02 | 0 |
| 7/08/2013 | 15.27 | 6.2 | 0.388 | 0 | 0.5 |
| 8/08/2013 | 15.36 | 133.1 | 7.829 | 0.01 | 6.2 |
| 9/08/2013 | 15.13 | 50.1 | 2.947 | 0.03 | 133.1 |
| 10/08/2013 | 15.11 | 0 | 0.000 | 0.07 | 50.1 |
| 11/08/2013 | 15.19 | 0 | 0.000 | 0.13 | 0 |
| 12/08/2013 | 15.24 | 0.2 | 0.013 | 0.21 | 0 |
| 13/08/2013 | 15.12 | 0 | 0.000 | 0.3 | 0.2 |
| 14/08/2013 | 15.03 | 0 | 0.000 | 0.4 | 0 |
| 15/08/2013 | 15.02 | 0 | 0.000 | 0.51 | 0 |
| 16/08/2013 | 14.97 | 0 | 0.000 | 0.62 | 0 |
| 17/08/2013 | 14.93 | 0.2 | 0.013 | 0.73 | 0 |
| 18/08/2013 | 15.08 | 0 | 0.000 | 0.83 | 0.2 |
| 19/08/2013 | 15.06 | 0 | 0.000 | 0.91 | 0 |
| 20/08/2013 | 15.06 | 0 | 0.000 | 0.96 | 0 |
| 21/08/2013 | 14.83 | 0 | 0.000 | 0.99 | 0 |
| 22/08/2013 | 14.69 | 0 | 0.000 | 1 | 0 |
| 23/08/2013 | 14.59 | 0 | 0.000 | 0.97 | 0 |
| 24/08/2013 | 14.59 | 0 | 0.000 | 0.92 | 0 |
| 25/08/2013 | 14.65 | 0 | 0.000 | 0.85 | 0 |
| 26/08/2013 | 14.73 | 0 | 0.000 | 0.77 | 0 |
| 27/08/2013 | 14.85 | 0 | 0.000 | 0.68 | 0 |
| 28/08/2013 | 15.00 | 0.2 | 0.013 | 0.58 | 0 |
| 29/08/2013 | 15.13 | 0.5 | 0.033 | 0.49 | 0.2 |
| 30/08/2013 | 15.41 | 0.5 | 0.033 | 0.39 | 0.5 |
| 31/08/2013 | 15.64 | 0 | 0.000 | 0.3 | 0.5 |
| 1/09/2013 | 15.71 | 0 | 0.000 | 0.22 | 0 |
| 2/09/2013 | 15.85 | 0 | 0.000 | 0.15 | 0 |
| 3/09/2013 | 16.01 | 0 | 0.000 | 0.09 | 0 |
| 4/09/2013 | 16.15 | 0 | 0.000 | 0.04 | 0 |
| 5/09/2013 | 16.37 | 0 | 0.000 | 0.01 | 0 |
| 6/09/2013 | 16.57 | 0 | 0.000 | 0 | 0 |
| 7/09/2013 | 16.75 | 0 | 0.000 | 0.01 | 0 |
| 8/09/2013 | 16.95 | 0.2 | 0.013 | 0.05 | 0 |
| 9/09/2013 | 17.05 | 0.6 | 0.038 | 0.1 | 0.2 |
| 10/09/2013 | 17.13 | 0 | 0.000 | 0.17 | 0.6 |
| 11/09/2013 | 17.07 | 0 | 0.000 | 0.27 | 0 |
| 12/09/2013 | 16.82 | 0 | 0.000 | 0.37 | 0 |
| 13/09/2013 | 16.73 | 0.2 | 0.013 | 0.48 | 0 |
| 14/09/2013 | 16.70 | 71.7 | 4.481 | 0.6 | 0.2 |
| 15/09/2013 | 16.78 | 4.2 | 0.263 | 0.71 | 71.7 |
| 16/09/2013 | 16.81 | 5.8 | 0.363 | 0.81 | 4.2 |
| 17/09/2013 | 16.84 | 585 | 36.563 | 0.89 | 5.8 |
| 18/09/2013 | 16.90 | 3.5 | 0.233 | 0.95 | 585 |
| 19/09/2013 | 16.78 | 0.2 | 0.013 | 0.99 | 3.5 |
| 20/09/2013 | 16.62 | 0 | 0.000 | 1 | 0.2 |
| 21/09/2013 | 16.43 | 4 | 0.267 | 0.99 | 0 |
| 22/09/2013 | 16.45 | 0 | 0.000 | 0.95 | 4 |
| 23/09/2013 | 16.74 | 0.4 | 0.027 | 0.89 | 0 |
| 24/09/2013 | 17.57 | 0 | 0.000 | 0.82 | 0.4 |
| 25/09/2013 | 17.70 | 0 | 0.000 | 0.74 | 0 |
| 26/09/2013 | 17.69 | 0 | 0.000 | 0.65 | 0 |
| 27/09/2013 | 17.55 | 0 | 0.000 | 0.56 | 0 |
| 28/09/2013 | 17.47 | 0 | 0.000 | 0.47 | 0 |
| 29/09/2013 | 17.43 | 0 | 0.000 | 0.37 | 0 |
| 30/09/2013 | 17.56 | 0.5 | 0.033 | 0.28 | 0 |
| 1/10/2013 | 17.68 | 0.5 | 0.031 | 0.2 | 0.5 |
| 2/10/2013 | 17.63 | 0 | 0.000 | 0.13 | 0.5 |
| 3/10/2013 | 17.30 | 0 | 0.000 | 0.07 | 0 |
| 4/10/2013 | 17.04 | 59.2 | 3.700 | 0.03 | 0 |
| 5/10/2013 | 17.17 | 1 | 0.063 | 0 | 59.2 |
| 6/10/2013 | 17.50 | 0 | 0.000 | 0 | 1 |
| 7/10/2013 | 17.75 | 0 | 0.000 | 0.03 | 0 |
| 8/10/2013 | 17.88 | 2.4 | 0.150 | 0.08 | 0 |
| 9/10/2013 | 18.02 | 0 | 0.000 | 0.15 | 2.4 |
| 10/10/2013 | 18.27 | 0 | 0.000 | 0.24 | 0 |
| 11/10/2013 | 18.49 | 2 | 0.125 | 0.34 | 0 |
| 12/10/2013 | 18.69 | 0 | 0.000 | 0.45 | 2 |
| 13/10/2013 | 18.86 | 0 | 0.000 | 0.57 | 0 |
| 14/10/2013 | 18.93 | 60.9 | 3.806 | 0.68 | 0 |
| 15/10/2013 | 18.78 | 0 | 0.000 | 0.78 | 60.9 |
| 16/10/2013 | 18.91 | 0 | 0.000 | 0.86 | 0 |
| 17/10/2013 | 18.98 | 0 | 0.000 | 0.93 | 0 |
| 18/10/2013 | 18.84 | 0 | 0.000 | 0.98 | 0 |
| 19/10/2013 | 18.81 | 0 | 0.000 | 1 | 0 |
| 20/10/2013 | 18.98 | 0 | 0.000 | 1 | 0 |
| 21/10/2013 | 19.08 | 0 | 0.000 | 0.97 | 0 |
| 22/10/2013 | 19.10 | 0 | 0.000 | 0.93 | 0 |
| 23/10/2013 | 19.14 | 70.5 | 4.700 | 0.88 | 0 |
| 24/10/2013 | 19.13 | 0 | 0.000 | 0.81 | 70.5 |
| 25/10/2013 | 19.08 | 4 | 0.250 | 0.72 | 0 |
| 26/10/2013 | 18.95 | 0 | 0.000 | 0.64 | 4 |
|  |  |  |  |  |  |

Table S4: Hurdle models assessing the influence of environmental variables: mean water temperature (°C), moon illumination, rainfall from that day (mm) or rainfall from the previous day (mm) on: a) presence-absence and b) abundance of sharks in Sydney Harbour. Random effects and offsets included. s = smoother term.

| Model | ΔAICc | Deviance explained (%) |
| --- | --- | --- |
| ~ s(temp) + s(total rain) + s(moon illumination) + s(total rain previous day) | 3.8 | 23.3 |
| ~ s(total rain) + s(moon illumination) + s(total rain previous day) | 71.4 | 0.87 |
| ~ s(temp) + s(moon illumination) + s(total rain previous day) | 2 | 23.3 |
| ~ s(temp) + s(total rain previous day) | 2.4 | 22.7 |
| ~ s(temp) | 0 | 22.6 |

**References**

1 Oh, B. Z. *et al.* Contrasting patterns of residency and space use of coastal sharks within a communal shark nursery. *Marine and Freshwater Research* **68**, 1501-1517 (2017).

2 Huveneers, C. *et al.* The influence of environmental parameters on the performance and detection range of acoustic receivers. *Methods in Ecology and Evolution*, doi:10.1111/2041-210X.12520 (2016).

3 R: a language and environment for statistical computing (R Foundation for Statistical Computing, Vienna, Austria, 2009).

4 Wood, S. N. Fast stable restricted maximum likelihood and marginal likelihood estimation of semiparametric generalized linear models. *Journal of the Royal Statistical Society: Series B (Statistical Methodology)* **73**, 3-36 (2011).

5 Wood, S. N. *Generalized Additive Models: An Introduction with R*. (Chapman and Hall/CRC, 2006).

6 Payne, N. L., Gillanders, B. M., Webber, D. M. & Semmens, J. M. Interpreting diel activity patterns from acoustic telemetry: the need for controls. *Marine Ecology Progress Series* **419**, 295-301 (2010).
